# Population and seascape genomics of a critically endangered benthic elasmobranch, the blue skate *Dipturus batis*

**DOI:** 10.1111/eva.13327

**Published:** 2021-12-07

**Authors:** Aurélien Delaval, Michelle Frost, Victoria Bendall, Stuart J. Hetherington, David Stirling, Galice Hoarau, Catherine S. Jones, Leslie R. Noble

**Affiliations:** ^1^ Faculty of Biosciences and Aquaculture Nord University Bodø Norway; ^2^ School of Biological Sciences University of Aberdeen Aberdeen UK; ^3^ Centre for Environment Fisheries and Aquaculture Science Lowestoft UK; ^4^ Marine Scotland Science Aberdeen UK

**Keywords:** blue skate, climate change, conservation, *Dipturus batis*, population genomics, seascape genomics

## Abstract

The blue skate (*Dipturus batis*) has a patchy distribution across the North‐East Atlantic Ocean, largely restricted to occidental seas around the British Isles following fisheries‐induced population declines and extirpations. The viability of remnant populations remains uncertain and could be impacted by continued fishing and by‐catch pressure, and the projected impacts of climate change. We genotyped 503 samples of *D. batis*, obtained opportunistically from the widest available geographic range, across 6 350 single nucleotide polymorphisms (SNPs) using a reduced‐representation sequencing approach. Genotypes were used to assess the species’ contemporary population structure, estimate effective population sizes and identify putative signals of selection in relation to environmental variables using a seascape genomics approach. We identified genetic discontinuities between inshore (British Isles) and offshore (Rockall and Faroe Island) populations, with differentiation most pronounced across the deep waters of the Rockall Trough. Effective population sizes were largest in the Celtic Sea and Rockall, but low enough to be of potential conservation concern among Scottish and Faroese sites. Among the 21 candidate SNPs under positive selection was one significantly correlated with environmental variables predicted to be affected by climate change, including bottom temperature, salinity and pH. The paucity of well‐annotated elasmobranch genomes precluded us from identifying a putative function for this SNP. Nevertheless, our findings suggest that climate change could inflict a strong selective force upon remnant populations of *D. batis*, further constraining its already‐restricted habitat. Furthermore, the results provide fundamental insights on the distribution, behaviour and evolutionary biology of *D. batis* in the North‐East Atlantic that will be useful for the establishment of conservation actions for this and other critically endangered elasmobranchs.

## 1 INTRODUCTION

Many elasmobranchs have experienced drastic population declines as a consequence of fishing pressure during the last century, representing a major conservation concern. Almost one‐third of elasmobranch species globally are threatened with extinction, yet nearly half remain too data‐deficient to be assessed (Dulvy et al., 2014, 2017; IUCN, 2021). Despite fishing restrictions, a large number are still caught as by‐catch, particularly in unregulated coastal and continental waters (Dulvy et al., 2017) where they can be of significant socio‐economic importance to local fisheries (Bendall et al., 2018; ICES, 2020). The *K*‐selected life history that most elasmobranchs exhibit exacerbates the impacts of exploitation; their characteristically slow growth, late‐onset maturity and relatively low reproductive output limit population recovery potential (Dulvy et al., 2017). In addition, evidence is mounting on the consequences of climate change for elasmobranch fitness (Di Santo, 2016; Dziergwa et al., 2019; Pistevos et al., 2015). For many data‐deficient elasmobranchs, instituting appropriate conservation actions requires a better understanding of their population structure and of their current and future realized niche in the face of environmental changes.

Elasmobranchs exhibit a range of life history traits that translate to different degrees of population structuring. Some species demonstrate high levels of gene flow across ocean basins (Lieber et al., 2020), while others are divided into smaller subpopulations with limited gene flow (Le Port & Lavery, 2012; Thorburn et al., 2018). A wide range of behaviours such as site fidelity and natal philopatry (Corrigan et al., 2018; Feutry et al., 2017; Pardini et al., 2001; Thorburn et al., 2018), long‐distance migrations (Blower et al., 2012; Cameron et al., 2018; Corrigan et al., 2018) and aggregating behaviour among closely related individuals (Lieber et al., 2020; Thorburn et al., 2018) can shape patterns of elasmobranch population connectivity and genetic diversity. In addition, environmental discontinuities such as bathymetric barriers (Le Port & Lavery, 2012) and temperature gradients (Griffiths et al., 2010) can influence species distributions and population connectivity, especially for less vagile species. The diversity and complexity of elasmobranch life histories are likely underappreciated due to issues such as taxonomic confusion (Iglésias et al., 2010) and misreporting of catches (ICES, 2020). Consequently, current conservation strategies that include marine protected areas (MPAs) have been suggested by some as oversimplified and ineffective (Dulvy et al., 2017; Dureuil et al., 2018), requiring more comprehensive species‐specific assessments.

Climate change represents a major threat to global biodiversity. In particular, climatic extremes such as maximum temperatures may lead to higher probabilities of local extinctions for species that are unable to disperse or adapt to these conditions (Román‐Palacios & Wiens, 2020). In the North‐East Atlantic Ocean, coastal waters are projected to experience temperature rises and acidification, and decreasing dissolved oxygen and salinity levels by the end of the century, while extreme oceanographic events are expected to increase in frequency and magnitude (MCCIP, 2020; Penny Holliday et al., 2020). Rising temperatures have already been associated with poleward distribution shifts for many species (Barton et al., 2016; Brattegard, 2011; Chaudhary et al., 2021; Perry et al., 2005) and have been linked to decreases in individual growth rates and fitness through the pejus effect (Morrongiello & Thresher, 2015). Behavioural changes have also been documented on a local scale, with some benthic elasmobranchs exploiting deeper thermal refugia (Perry et al., 2005). However, species that are more sedentary in nature may not be capable of undertaking spatial distribution shifts; in these cases, survival may depend upon physiological adaptation to a changing environment. For marine elasmobranchs, the projected environmental changes are likely to incur important physiological costs, particularly in relation to osmoregulation and acid‐base regulation to maintain homeostasis. While some elasmobranchs have adapted strategies to cope with environmental extremes (Dziergwa et al., 2019; Heinrich et al., 2014), others are likely to suffer greater losses in individual fitness (Di Santo, 2016; Pistevos et al., 2015).

For nonmodel species that cannot be studied in situ or experimentally, novel molecular approaches in the era of next‐generation sequencing (NGS) can provide insights into the structure and local adaptation of wild populations. Ideally, the assembly and annotation of full genomes would provide a functional basis for genomic investigations of a species. However, genome assembly remains prohibitively costly and resource heavy to address urgent conservation questions at the scale of populations, especially given that elasmobranch genomes can be large and complex (Hara et al., 2018). Reduced‐representation sequencing (RRS) methods provide an alternative approach, whereby thousands of genome‐wide single nucleotide polymorphisms (SNPs) can be examined in the absence of a reference genome (Andrews et al., 2016). These generate high‐resolution data to estimate genetic differentiation even when sample sizes are small (Willing et al., 2012), which is an advantage for studies on rare species that rely on opportunistic sampling. Genome‐wide SNPs can also be used to estimate effective population size (*N*_e_), a theoretical estimator of population size after accounting for genetic drift, which is often used in conservation genetics (reviewed in Allendorf et al., 2013). Furthermore, genotype–environment studies have taken a leap forward with the arrival of NGS methods. Landscape (or seascape) genomics combines genomic and environmental data to investigate how genetic structuring may be driven by environmental variables, and can reveal candidate genes under selection in certain environmental conditions (Balkenhol et al., 2017; Riginos et al., 2016; Roffler et al., 2016).

In this study, we used a population and seascape genomics approach to investigate patterns of population structuring, abundance and local adaptation in a critically endangered elasmobranch, the blue skate *Dipturus batis* (Linnaeus, 1758). *D. batis* has only recently received species status after morphological and genetic investigations distinguished it from the parapatric flapper skate *D. intermedius* (Griffiths et al., 2010; Iglésias et al., 2010). Both species have become a conservation priority as a result of population declines and range restrictions, but are still caught as by‐catch despite an EU landing ban (Ellis et al., 2016). In addition to the continual threat posed by fisheries‐induced mortality, the ability of the skates to adapt to environmental changes has become a pertinent question; *D. batis* currently exploits a narrower thermal niche than *D. intermedius* (Frost et al., 2020) and consequently may respond differently to ocean warming. Using samples collected from the widest available extent of *D. batis’* range, obtained through a combination of research surveys and samples of opportunity, we applied a RRS approach (DArTseq^TM^; Kilian et al., 2012) to (i) assess the level of gene flow and levels of genetic diversity among extant *D. batis* populations, (ii) estimate their effective population sizes and (iii) identify potential signals of selection in relation to environmental conditions. Assessment of *D. batis*’ population structure and the potential consequences of environmental change on its ecological niche are necessary to define current and future spatial management units, and may help identify areas that may qualify for additional protection. The threat of continued fishing mortality and uncertain effects of environmental change, combined with the challenge presented by data deficiency, are characteristics that *D. batis* shares with many endangered elasmobranchs. It is hoped that our primarily molecular approach will effectively address important knowledge gaps for *D. batis* and that it may be extended to other elasmobranchs of conservation concern.

## 2 MATERIALS AND METHODS

### 2.1 Study species

The blue skate *Dipturus batis* is one of two rajids classified as Critically Endangered by the IUCN (Dulvy et al., 2006) formerly belonging to the common skate species complex (*D. batis* complex). The two common skate species, which co‐occur in parts of their North‐East Atlantic range (Frost et al., 2020), were recently differentiated into the smaller‐bodied blue skate (*D. batis*) and the larger‐bodied flapper skate (*D. batis*) based on morphological and genetic differences (Griffiths et al., 2010; Iglésias et al., 2010). At present, the confirmed geographic range of *D. batis* extends from the Celtic Sea to north of Orkney, with higher densities occurring in the Celtic Sea and Rockall (Frost et al., 2020; Griffiths et al., 2010), while their occurrence has recently been confirmed in Iceland (Bache‐Jeffreys et al., 2021).

### 2.2 Sample collection

**FIGURE 1:**
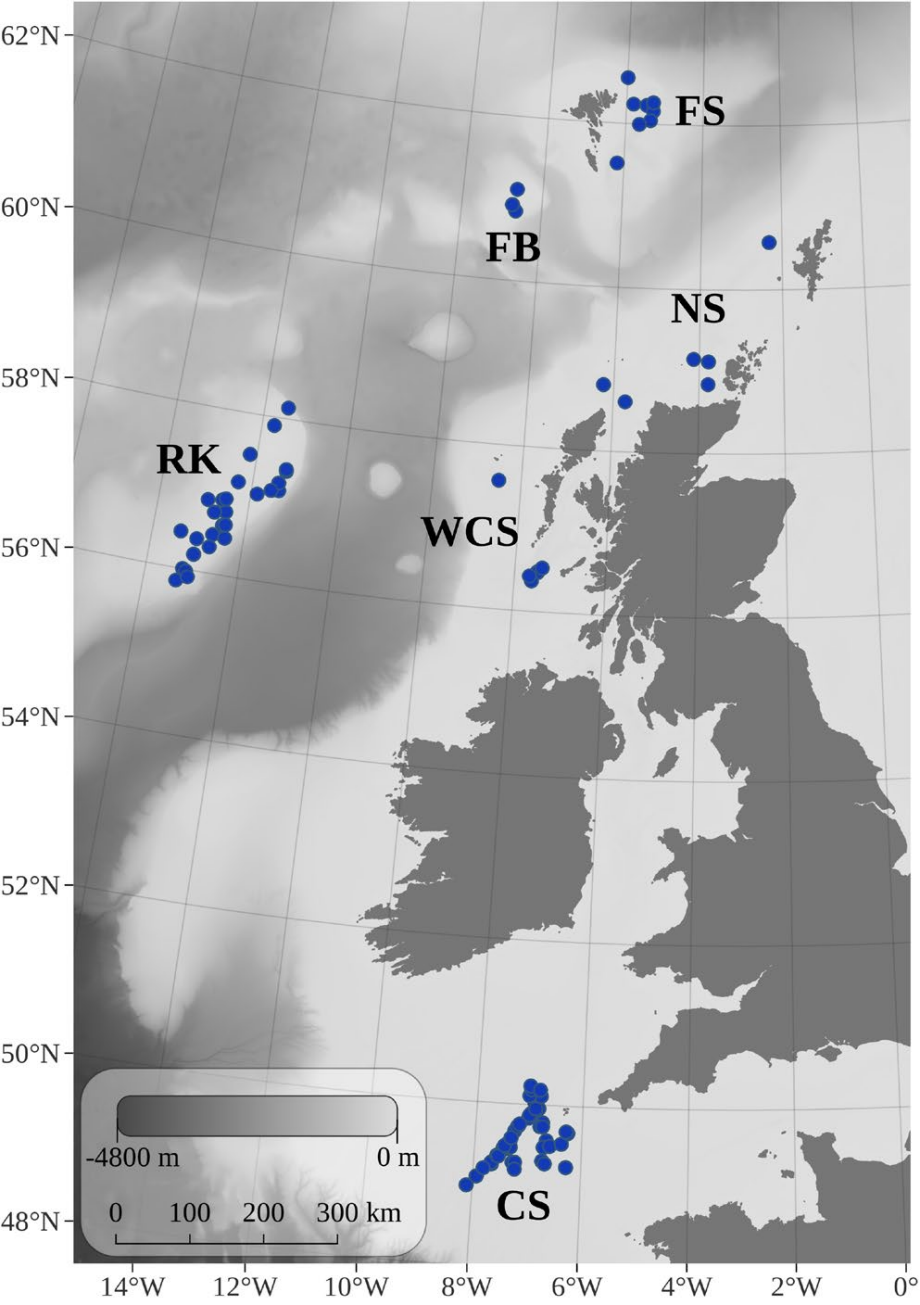
Sampling locations across the North‐East Atlantic Ocean for 503 blue skate *Dipturus batis* that were used for population genomic analyses. Site names are abbreviated for the Celtic Sea (CS), West Coast Scotland (WCS), Northern Scotland (NS), Rockall (RK), the Faroe Bank (FB) and the Faroe Shelf (FS)

Samples of *D. batis* were primarily obtained from fishery‐dependent surveys conducted in the Celtic Sea during the autumn from 2011 to 2017 (Figure S1), and from fishery‐independent surveys conducted along the Scottish coast and at Rockall. Faroese samples, obtained opportunistically from fisheries‐independent surveys and the commercial fishing vessel ‘Sandshavið’, were donated by the Faroe Marine Institute. Individuals were identified as *D. batis* based on the morphological characteristics described by Iglésias et al. (2010), namely body size, eye colour, dorsal patterning and interdorsal space. In addition, 113 of the individuals included in this study (102 from the Celtic Sea, five from Northern Scotland and six from Rockall) were genetically validated as *D. batis* using diagnostic microsatellite markers by Frost et al. (2020). Sex and length (cm) data were collected from all samples except those from the Faroe Bank. Fin or muscle tissue samples were collected and stored in 96% ethanol or RNAlater^®^. A sample size of 4–6 individuals reportedly provides sufficient power for resolving population genomic structure when over 1000 biallelic markers are used (Willing et al., 2012). To ensure sufficient power in our study, we selected at least 10 individuals from different geographic areas where available, across the narrowest temporal range possible to minimize temporal structuring of our sample set. A total of 564 samples were selected for genomic analysis, from six locations: the Celtic Sea, the Scottish West Coast, Northern Scotland, Rockall, the Faroe Bank and the Faroe Shelf (Figure 1, Table 1).

**TABLE 1:** Overview of sample sizes of *Dipturus batis* selected for genomic analysis from six geographic areas, and resulting sample sizes after SNP and sample filtering

		Sample size			Biological characteristics	
Locality	Year	Initial	Postfiltering	After removal of close relatives	No. of males (size range)	No. of females (size range)
Celtic Sea (CS)	2011–2017^a^	417	387	379	186 (66–138)	201 (69–148)
West Coast Scotland (WCS)	2012–2013	33	18	18	11 (28–108)	7 (21–106)
North Scotland (NS)	2013, 2019	14	9	9	2 (45–103)	7 (32–79)
Rockall (RK)	2012–2013	80	70	69	28 (33–123)	42 (30–127)
Faroe Shelf (FS)	2019	10	10	10	4 (132–152)	6 (46–152)
Faroe Bank (FB)	2019	10	9	8	NA (90–145)	
Total		564	503	493		

Note

The number of males and females after filtering (*N* = 503) is shown together with their size range (length in cm).

^a^ Details of the Celtic Sea survey are shown in Figure S1.

### 2.3 DNA extraction and genotyping

Genomic DNA was extracted using a DNeasy^®^ Blood & Tissue Kit (Qiagen). DNA concentrations were quantified on a Qubit fluorometer (Thermo Fisher Scientific) and adjusted to 10–60 ng/µl in preparation for sequencing. To assess the quality of DNA, we performed a mock digest of the samples in CutSmart^®^ Buffer (New England Biolabs) for 2 h at 37°C, and resolved all samples on a 0.8% TAE electrophoresis gel. In addition, two samples from each locality were visualized on a Genomic DNA ScreenTape^®^ for a more detailed assessment of DNA quality. Genotyping was performed by Diversity Arrays Technology (DArT Pty. Ltd., Canberra, Australia) using DArTseq^TM^ following standard protocols as described in Kilian et al. (2012). DArTseq^TM^ combines complexity reduction methods and NGS, and is optimized for each organism. Based on tests of several enzyme combinations for complexity reduction, DArT Pty. Ltd. applied the restriction enzyme combination *PstI* and *SphI* on the samples. Samples were sequenced (single read) on an Illumina^®^ HiSeq^®^ 2500, generating approximately 1.5 million sequences per sample. Sequences were processed using proprietary DArT Pty. Ltd. analytical pipelines. Twelve samples were identified as nontarget species (data not shown) and were removed from the data set before processing of raw sequences was repeated. This generated data for 17,620 sequences of ~69‐bp length, each containing a SNP.

### 2.4 Data filtering

Ten samples were not reported by DArT Pty. Ltd. due to poor sample quality, and an additional sample was removed due to suspected genotyping error, as determined by visually scanning the raw data. SNPs were further filtered based on a call rate of 80%, and when duplicate loci were present, only the locus with the highest call rate was retained. After this step, the proportion of scored loci per sample was assessed, and all samples were considered to have a sufficiently high score rate to be retained (>88%). Monomorphic loci and loci with low minor allele frequencies (MAF < 0.05) across all samples were identified using *adegenet* (v 2.1.2, Jombart, 2008; Jombart & Ahmed, 2011), as implemented in R (v 3.6.2, R Core Team, 2019), and subsequently removed. Because human error could lead to sampling an individual multiple times or to contamination during molecular laboratory work, we looked for duplicate samples based on a threshold of 700 mismatching loci (roughly 10% of remaining loci) using the R package *CKMRsim* (Anderson, https://doi.org/10.5281/zenodo.820162). Where duplicates were found (i.e. >90% genetically identical), the sample with the highest score rate was retained. Next, we tested for conformation of loci to the Hardy–Weinberg proportions using the R package *pegas* (v 0.12, Paradis, 2010), performing an exact test based on Monte Carlo permutation of alleles (Guo & Thompson, 1992) with 1000 replicates for each of the six sampling locations and for the entire dataset (Table S2). After applying the false discovery rate (FDR) correction method of Benjamini and Hochberg (1995), loci were removed if they significantly deviated from Hardy–Weinberg proportions (at a significance threshold of *α* = 0.05) in at least two sampling locations. We then tested for linkage disequilibrium among loci using the R package *snpStats* (v 1.36.0; Clayton, 2019) and removed one locus from each pair for which *R* ^2^ > 0.80. Following these filtering steps (summarized in Table S1), the resulting data set contained 503 individuals genotyped at 6,350 loci (Table 1, Figure 1).

### 2.5 Finding related individuals

Because related pairs of individuals may introduce a bias in population genomic analyses, particularly when sample sizes are small, we looked for first‐order (e.g. parent–offspring, full‐sibling) and second‐order (e.g. half‐sibling) relatives in our data set and removed one individual of each related pair for downstream analyses. Identifying related individuals also allowed us to observe any patterns of family structuring and habitat use. Related individuals were identified using *CKMRsim*, which simulates related pairs of individuals based on observed allele frequencies using a Monte Carlo approach. Using *CKMRsim*, we calculated the false‐positive and false‐negative rates at different log‐likelihood thresholds for each pairwise hypothesis test involving parent–offspring (PO), full‐sibling (FS), half‐sibling (HS) and unrelated (U) relationship categories. Due to the large number of pairwise comparisons in relationship testing (503 samples imply 126,253 pairwise tests), this approach allowed us to identify appropriate log‐likelihood thresholds when performing the relationship tests. Following *CKMRsim* recommendations, we aimed for a false‐positive rate threshold of 100 times smaller than the reciprocal of the number of comparisons made (i.e. FPR < 7.92 × 10^−8^). We identified 10 related pairs and removed one individual from each pair such that population genomic analyses involved 493 individuals (Table 1).

### 2.6 Population structure

Population‐ and locus‐wide summary statistics were obtained using GenAlEx (v 6.5, Peakall & Smouse, 2006, 2012) with the exception of allelic richness, which was estimated using the R package *PopGenReport* (Adamack & Gruber, 2014). Spatial population structure was assessed using three approaches. First, we employed a Bayesian clustering algorithm using STRUCTURE (v 2.3.4, Pritchard et al., 2000). We used an admixture model with correlated allele frequencies, a burn‐in length of 300,000 (more than enough to reach convergence) followed by 500,000 MCMC, and performed five iterations for each prior subpopulation number *K* (ranging from *K* = 1 to *K* = 6). In order to avoid impractically long computation times and potential biases resulting from imbalanced sample sizes (Wang, 2017), we randomly subsampled 10 individuals from the Celtic Sea, West Coast Scotland and Rockall, and included all samples from North Scotland (*N *= 9), the Faroe Shelf (*N *= 10) and the Faroe Bank (*N *= 8), such that the total sample size for STRUCTURE analysis was 57. The most likely value of *K* was estimated using the delta‐*K* method of Evanno et al. (2005) in STRUCTURE Harvester (Earl & VonHoldt, 2012), and summary plots for each *K* were produced using CLUMPAK (Kopelman et al., 2015). In order to justify the pooling of samples from different years within sample sites, and to ensure that no fine‐scale structure would go undetected by subsampling the data for the STRUCTURE analysis, we tested for population structure among all 493 individuals using fastSTRUCTURE (Raj et al., 2014), a more rapid analysis that uses approximate inference of the Bayesian model in STRUCTURE. For fastSTRUCTURE, we used a simple prior model with a seed of 1000, and tested for *K* = 1 through *K* = 6. The output files were summarized using StructureSelector (Li & Liu, 2018; Figures S3 and S4). In addition, we performed exploratory STRUCTURE runs to test for genomic heterogeneity among sampling years for the 379 Celtic Sea samples. We found no evidence of genetic heterogeneity in the Celtic Sea between 2011 and 2017 (Figures S5 and S6).

Second, spatial genomic structure was assessed for all individuals (filtered dataset, *N *= 493) using a discriminant analysis of principal components (DAPC) in *adegenet*. Missing data at 37,939 loci (i.e. 1.2% of loci among all samples) were replaced with the mean allele frequencies across all samples. Cluster identification was performed using the *find*.*clusters* function, with the optimal number of clusters evaluated using the Bayesian information criterion (BIC). Two DAPC plots were produced: one in which the prior grouping of individuals was based on the evaluated number of clusters, and one with prior groupings based on the six predefined geographic locations.

Third, we performed a principal component analysis (PCA) with the R function *prcomp*. We used the prefiltered data set of 503 individuals, as the first two principal components from the PCA would later be used as covariates for each individual in our seascape genomics analysis, in which we included all 503 individuals. Since the function does not allow for any missing data, we utilized 3 540 loci with a call rate of 100%.

Overall F‐statistics (*F*_IS_, *F*_ST_) and pairwise *F*_ST_ (Weir & Cockerham, 1984) between sampling locations were estimated with the R implementation of *GenePop* (v 1.1.3, Rousset, 2008). *GenePop* was also used to perform overall and pairwise tests of genic differentiation, testing the null hypothesis that all alleles are drawn from the same distribution in all populations. Here, we applied an exact G test (Fisher's method), using 1 000 dememorizations, 100 batches and 1 000 iterations per batch.

### 2.7 Effective population size

The effective population size (*N*_e_) is a theoretical estimator of population size after accounting for genetic drift that is useful in conservation genetics as it reflects the additive genetic variation, or evolutionary potential, of wild populations (reviewed in Allendorf et al., 2013). We estimated contemporary *N*_e_ for each sampling location and for each putative population (inferred from the preceding analyses) using the linkage disequilibrium (LD) estimator (Hill, 1981; Waples, 2006; Waples & Do, 2010) in NeEstimator (v 2.1, Do et al., 2014). The estimate assumed random mating and was performed at critical values (i.e. MAF at which alleles should be excluded) of 0.05, 0.02 and 0.01. Confidence intervals were obtained using the jackknife‐over‐individuals method.

### 2.8 Seascape genomics and candidate loci under selection

We tested for associations between allele frequencies and environmental variables using Samβada (v 0.8.1; Joost et al., 2007; Stucki et al., 2017), software for landscape (or seascape) genomic analysis of large data sets that uses multiple logistic regressions to estimate the probability of a genetic marker being present given a set of environmental conditions. We obtained environmental variables that were representative of the skate's primarily epibenthic habitat. Monthly means for four physical (temperature, mixed layer depth, salinity and current velocity) and seven biogeochemical (chlorophyll, dissolved oxygen, nitrate, phosphate, pH, primary production and light attenuation) variables were obtained from the Ocean Physical and Biogeochemical Reanalysis (NWSHELF_MULTIYEAR_PHY_004_009 and NWSHELF_MULTIYEAR_BGC_004_011) data products, available from the Copernicus Marine Service (https://marine.copernicus.eu/). Information was extracted for the near‐bottom depth layer for each variable (where relevant, i.e. excluding mixed layer depth and light attenuation) and for the 12 months preceding each individual's sampling date at a spatial resolution of ~7 km; these criteria were selected in order to obtain data representative of the skates’ year‐round environment and to minimize any seasonal effects from when skates were sampled. Overall mean, maximum and minimum values for each of these variables were retained for our seascape genomics analysis. In attempting to characterize the skates’ year‐round environment, such an approach assumed that the skates remained near their sampled locations in the 12 months prior to being sampled. Bottom depths for each sample were obtained from the EMODnet Bathymetry Consortium (2020). Five individuals from the Celtic Sea had erroneous or missing sample site information and were excluded from the analysis. Seven samples collected at the Faroe Bank by commercial fishermen also lacked precise sampling metadata, but are known to have been collected around late August–early September 2019 from the south‐western part of the Faroe Bank shallower than 200 m (Faroe Marine Institute pers. comm.). To obtain environmental variables for these samples, the statistical rectangles from ICES subdivision 5.b.2 were used to subset the bathymetric layer. This subset was then binarized (> / <200 m) and the centroid for the polygon above 200‐m depth used as spatial coordinates for those records.

We performed a PCA to characterize the environmental variation among sites and to identify those variables responsible for this variation. We calculated Spearman's correlation coefficients among all variables, to allow for removal of those showing collinearity from the multiple logistic regression. In Samβada, the frequencies of alleles at each locus were tested for associations with latitude, longitude, depth, and the 11 physical and biogeochemical variables (means, minima and maxima). To account for population structure, we employed a multivariate model, taking the first two principal components from a PCA performed on the genomic data set as covariates for each individual. We computed P‐values based on G and Wald scores for each test, and corrected for type I error from multiple comparisons using the Bonferroni correction at thresholds of 0.05 and 0.01.

As an additional test for outlier loci, we implemented a Bayesian outlier detection method on all samples using BayeScan (v 2.1; Foll & Gaggiotti, 2008), which identifies loci for which allele frequencies in the defined subpopulations deviate significantly from those of the total gene pool (i.e. all populations). We used BayeScan's default parameters (i.e. 20 pilot runs of length 5 000 and an additional burn‐in length of 50,000) and applied a q‐value threshold of 0.05. Outlier loci from both approaches could therefore be compared. The sequences containing the SNPs detected in BayeScan and in our seascape genomics analysis were BLASTed on NCBI (NCBI, 1988) to identify functional sequences. The blastn function was used to allow for comparisons across species, given the poorly annotated nature of elasmobranch genomes. We reported the top five BLAST hits with an E‐value <0.01.

## 3 RESULTS

### 3.1 Sampled individuals

Sample sizes reflected sampling effort and corresponded to anecdotal reports of *D. batis* abundance. Most samples were obtained from the Celtic Sea and Rockall during scientific surveys (Table 1). Males and females were collected at all sites, and a higher number of females was collected overall. Sex was not recorded for the Faroe Bank skates. A broad size range of individuals was collected for both sexes (ranging from 21 to 152 cm), representing a mix of juveniles and adults at most sites based on estimated ages at first maturity for *D. batis* complex at 115 cm and 125 cm for males and females, respectively (McCully et al., 2012).

### 3.2 Related individuals

The final panel of 6 350 SNPs was very informative for the detection of related individuals among 503 samples of *Dipturus batis*. False‐positive rates were considerably lower than our defined threshold for all six pairwise relationship tests (FPR ≤5.62 × 10^−56^ in each case; Table S3, Figure S2). We identified 10 related pairs, including one full‐sibling pair and seven half‐sibling pairs from the Celtic Sea, one half‐sibling pair from Rockall and one half‐sibling pair from the Faroe Bank (Table 2). All related pairs were therefore found at the same locality, and in four of these cases, the pair was collected in the same haul (i.e. at the same time and place).

**TABLE 2:** Full‐sibling (FS) and half‐sibling (HS) pairs of blue skate *Dipturus batis* and their biological and sampling details. Note that Faroe Bank samples were obtained from a fishing vessel for which some sampling details are lacking

Relationship	Locality	ID number	Sampled date	Length (cm)	Sex	Latitude (decimal)	Longitude (decimal)
FS	Celtic Sea	2015‐16‐240	24.09.2015	121	M	49.317	−6.731
	Celtic Sea	2017‐C10‐4‐16	26.10.2017	122	F	49.197	−7.815
HS	Celtic Sea	2017‐C11‐2‐09	26.10.2017	94	F	49.092	−7.933
	Celtic Sea	2017‐C09‐3‐06	26.10.2017	84	F	49.270	−7.673
HS	Celtic Sea	2017‐C14‐21‐07	30.10.2017	97	M	50.225	−7.008
	Celtic Sea	2017‐C14‐21‐10	30.10.2017	115	M	50.225	−7.008
HS	Celtic Sea	2017‐C13‐20‐04	30.10.2017	113	M	50.178	−6.983
	Celtic Sea	2017‐C13‐20‐05	30.10.2017	129	M	50.178	−6.983
HS	Celtic Sea	2017‐C02‐15‐10	29.10.2017	120	M	50.105	−6.800
	Celtic Sea	2014‐80	17.09.2014	117	M	49.952	−6.835
HS	Celtic Sea	2015‐16‐242	24.09.2015	74	M	49.317	−6.731
	Celtic Sea	2017‐C05‐11‐06	29.10.2017	78	F	49.725	−7.216
HS	Celtic Sea	2015‐18‐253	24.09.2015	133	M	49.284	−6.682
	Celtic Sea	2011–279	24.08.2011	123	F	49.967	−6.850
HS	Celtic Sea	2011–271	24.08.2011	132	F	49.967	−6.850
	Celtic Sea	2011–308	24.08.2011	116	M	49.967	−6.850
HS	Rockall	1413S−125	24.10.2013	74	M	56.610	−14.444
	Rockall	1413S−126	24.10.2013	112	M	56.610	−14.444
HS	Faroe Bank	Sandshavið‐F1	2019	90	NA	NA	NA
	Faroe Bank	Sandshavið‐F2	2019	102	NA	NA	NA

### 3.3 Genetic diversity

Patterns of genetic diversity were generally comparable across sites; however, samples from Rockall exhibited lower genetic diversity (Table 3). Allelic richness, which unlike number of alleles corrects for differences in sample size, was lower at Rockall. Although higher at both Faroese sites, the Faroese samples had a slightly lower allelic richness when compared to the three British sites. The fixation index (*F*_IS_ = 1 − (*H*_o_/*H*_e_)), otherwise known as the inbreeding coefficient, was generally low across sites, but was higher at Rockall and West Coast Scotland. Negative values of *F*_IS_ for individuals from North Scotland, Faroe Bank and Faroe Shelf corresponded to a slightly higher‐than‐expected level of heterozygosity at these sites, whereas the opposite was true of the Celtic Sea, Rockall and West Coast Scotland. There was no evidence for the presence of unique loci at any of the sites, with no private alleles detected.

**TABLE 3:** Mean genomic summary statistics for *Dipturus batis* overall and across six sampling locations

Locality	*N*	*N*_a_	*H*_o_	*H*_e_	*F*	PA	*A*_r_
CS							
Mean	375.107	2.000	0.284	0.296	0.045	0	1.662
SE	0.137	0.000	0.002	0.002	0.002		
RK							
Mean	67.747	1.974	0.251	0.264	0.062	0	1.590
SE	0.040	0.002	0.002	0.002	0.002		
NS							
Mean	8.925	1.906	0.292	0.283	−0.034	0	1.646
SE	0.004	0.004	0.002	0.002	0.004		
WCS							
Mean	17.503	1.968	0.269	0.287	0.061	0	1.648
SE	0.015	0.002	0.002	0.002	0.004		
FB							
Mean	7.928	1.861	0.288	0.274	−0.050	0	1.625
SE	0.005	0.004	0.003	0.002	0.004		
FS							
Mean	9.902	1.900	0.277	0.276	−0.010	0	1.629
SE	0.006	0.004	0.002	0.002	0.004		
Overall							
Mean	81.185	1.935	0.277	0.280	0.014	0	
SE	0.682	0.001	0.001	0.001	0.001		

Note

Sample sizes (*N*), number of alleles (*N*_a_), observed (*H*_o_) and expected (*H*_e_) heterozygosity, fixation index (*F*) and number of private alleles (PA) are shown, as reported using GenAlEx (v 6.5, Peakall & Smouse, 2006, 2012). Mean allelic richness (*A*_r_) is also shown, as reported by *PopGenReport* (Adamack & Gruber, 2014).

### 3.4 Spatial population structure

Overall, the results suggest a clear barrier to gene flow between Rockall and all other sites, with Rockall demonstrating significant genomic differentiation across all analyses. There was high gene flow among British continental shelf sites (CS, WCS and NS), whereas gene flow occurred to a more limited extent between the British shelf and the Faroese sites; results of the different analyses led to different conclusions regarding the degree of genomic differentiation among Faroese and British skates.

**FIGURE 2:**
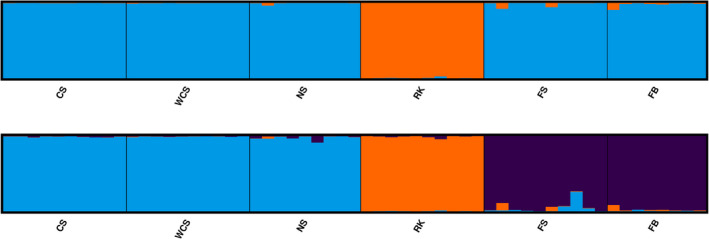
Results from the Bayesian clustering algorithm implemented in STRUCTURE, visualized using CLUMPAK for *K* = 2 (top) and *K* = 3 (bottom), for 57 *Dipturus batis* samples collected from the Celtic Sea (CS), West Coast Scotland (WCS), North Scotland (NS), Rockall (RK), the Faroe Shelf (FS) and the Faroe Bank (FB). Each individual is represented by a vertical line with the proportion of assignment to a cluster indicated by two or three colours

Results of the Bayesian clustering analysis implemented in STRUCTURE suggested that the most likely number of clusters was 2, as per the delta‐*K* method of Evanno et al. (2005). The clustering clearly separated Rockall skates from the rest (Figure 2). When visualizing the output of *K* = 3, which had only a slightly lower mean log probability than *K* = 2 (Figure S7), a third cluster consisting of Faroese skates was identified, with some proportion of assignment to the British cluster for a few samples from the Faroe Shelf (Figure 2). Similar results were obtained in fastSTRUCTURE (Figures S3 and S4).

**FIGURE 3:**
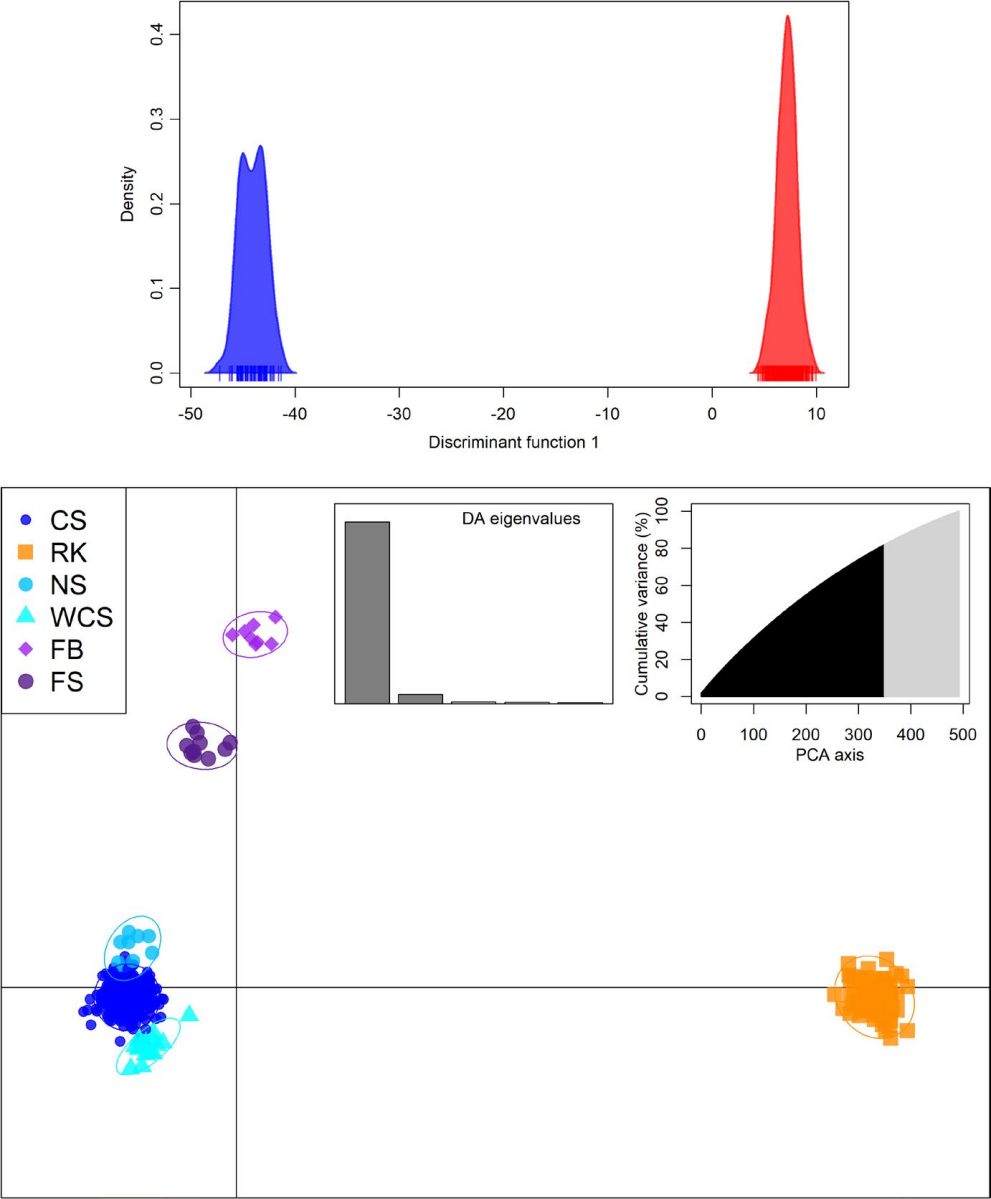
Discriminant analysis of principal component (DAPC, *adegenet*) plots depicting the variation among 493 *Dipturus batis* samples genotyped across 6 350 SNPs. Top: variation between two clusters inferred using *find*.*clusters*, where the blue (left) cluster contains all 69 samples from Rockall and the red (right) cluster contains the remaining 424 samples from the UK and Faroe Island sites. Bottom: variation among samples grouped by their sampling locations, with 95% inertia ellipses shown for each group. Site names are abbreviated for the Celtic Sea (CS), West Coast Scotland (WCS), Northern Scotland (NS), Rockall (RK), the Faroe Bank (FB) and the Faroe Shelf (FS)

The *find*.*clusters* function in the discriminant analysis of principal components (DAPC) suggested an optimal number of two clusters, based on the lowest BIC score after retaining all 493 principal components (Figure S9). Results were plotted using these inferred clusters and based on the six sampling locations using the first 350 principal components (explaining 82% of the observed variance). For the two inferred clusters, all 69 individuals from Rockall formed one cluster while the remaining 424 samples were clearly differentiated into a second cluster, summarized by only one discriminant function (Figure 3).

When grouped by sampling location, the same distinction between Rockall and the rest of the samples could be seen, summarized most informatively by the first of five eigenvalues (Figure 3). However, in this case, samples from the Faroe Bank and the Faroe Shelf could be distinguished from all other sites, and from each other.

In order to assess the influence of loci under selection and the level of potentially adaptive population structure, DAPC was repeated using only the 21 outlier loci identified in BayeScan, and with a neutral data set excluding these 21 loci. Despite weaker clustering that could be expected with the small number of loci, the DAPC still revealed genomic differentiation across the putatively adaptive loci (Figure S10), while the removal of these 21 loci from the total dataset did not affect the results (Figure S11). The PCA also showed a clear separation between samples from Rockall and the remaining sites; however, the variation explained by each principal component was low (≤ 1.2%, Figures S12 and S13).

Results of the G test suggested significant genic differentiation across samples overall (*p* < 0.001), and between all pairwise site comparisons with Rockall (*F*_ST_>0.038, *p* < 0.001, Table 4). Overall, *F*_ST_ and *F*_IS_ were low (*F*_ST_ = 0.026 and *F*_IS_ = 0.043). The G tests and F‐statistics were repeated after grouping individuals into three putative populations: British Shelf, Rockall and Faroe Islands. A test with this grouping was performed to statistically investigate the suspected isolation of Faroese skates from those on the continental shelf, as indicated by the STRUCTURE and DAPC results; the proximity of closely related individuals, tagging results reported by Bendall et al. (2018) and the depth range where *D. batis* was found suggest a plausible barrier to gene flow across the deep Faroe–Shetland channel for a species that is apparently site‐attached to shelf areas. After grouping, the overall G test indicated a significant genic differentiation overall and between all three pairs of sites (*p* < 0.001), and there was a slight increase in overall *F*_ST_ and *F*_IS_ (*F*_ST_ = 0.032 and *F*_IS_ = 0.044).

**TABLE 4:** Pairwise *F*_ST_ for *Dipturus batis* among six sites

	CS	RK	NS	WCS	FB	FS
CS	—					
RK	**0.0383**	—				
NS	0.0004	**0.0425**	—			
WCS	0.0013	**0.0420**	0.0003	—		
FB	0.0123	**0.0444**	0.0122	0.0133	—	
FS	0.0082	**0.0449**	0.0083	0.0089	0.0086	—

Note

Values in bold indicate significant genomic differentiation (*p* < 0.05) from pairwise *G* tests.

### 3.5 Effective population sizes

Effective population sizes (*N*_e_) were estimated for each sample site and after grouping samples into the three putative populations. For the former, sample sizes were too small and *N*_e_ could not be estimated for some sites (Table S4). For the latter, the British Shelf population had the highest *N*_e_ (ca. 21,000, Table 5), whereas *N*_e_ for Rockall was as low as half of this (estimates ranging from ca. 11,300 to 19,000). The Faroese skates demonstrated the lowest levels of *N*_e_ (estimates ranging from ca. 2 300 to 3 500). In some cases, estimates reached infinity, probably as a result of small sample sizes (Marandel et al., 2019) rather than very large population sizes.

**TABLE 5:** Estimates of effective population size (*N*_e_) for *Dipturus batis* grouped into three putative population units, using the linkage disequilibrium method in NeEstimator

Population	*N*	Crit = 0.05	Crit = 0.02	Crit = 0.01
British Shelf	406	21,068 (17,141–27,313)	21,015 (17,110–27,213)	21,010 (17,128–27,150)
Rockall	69	11,299 (3 903–∞)	14,475 (3 810–∞)	18,983 (3 943–∞)
Faroe Islands	18	2 362 (1 362–8 824)	3 501 (1 798–63,597)	3 501 (1 798–63,597)

Note

Estimates are shown for three critical values (Crit = 0.05, 0.02 and 0.01), and 95% confidence intervals by jackknifing over individuals are shown in parentheses. Sample sizes (*N*) are also shown.

### 3.6 Seascape genomics and candidate loci under selection

**FIGURE 4:**
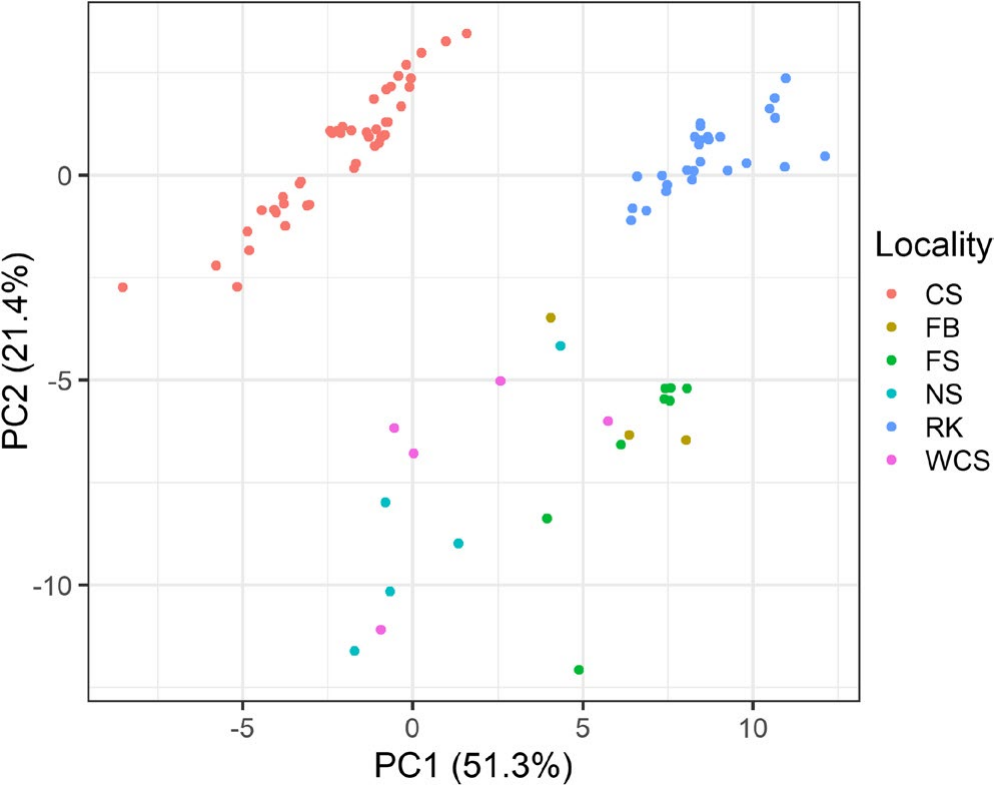
Principal component analysis depicting the variation among sites based on 34 environmental variables. Site names are abbreviated for the Celtic Sea (CS), West Coast Scotland (WCS), Northern Scotland (NS), Rockall (RK), the Faroe Bank (FB) and the Faroe Shelf (FS)

The PCA showed a clear differentiation among sites based on all 34 environmental variables. The first two principal components (PCs) explained 72.7% of the environmental variation among sites (Figure 4), with all variables contributing to at least one of these PCs (Figure S14). Overall, there was a clear difference between southern (Celtic Sea) and northern (all other sites) sites across PC1 and PC2, whereas a distinction could also be made between ‘offshore’ (Celtic Sea and Rockall) and ‘inshore’ (Scottish and Faroese, also including Faroe Bank) sites across PC2. The variation among sites was complex, but a general pattern could be seen where the Celtic Sea was warmer, more saline and more acidic, while Rockall was the deepest of the sites (up to 385 m deep; Table 6). There was a strong correlation between primary productivity and chlorophyll concentration and between nitrate and phosphate concentrations (Spearman's correlation coefficients >0.9). Removing one variable from each collinear pair did not influence the results of our multiple logistic regressions, and actually reduced the environmental variation explained in the PCA. Therefore, we report results of logistic regression using all 34 environmental variables.

**TABLE 6:** Overview of environmental characteristics for each site, showing mean and range (in parentheses) across monthly means obtained for 12 months prior to sampling date

Locality	Year	Depth (m)	BT (°C)	MLD (m)	Sal (PSU)	Cur (ms^−1^)	Chl (mg m^−3^)	O_2_ (mmol m^−3^)	${\text{NO}}_{3}^{‐}$ (mmol m^−3^)	${\text{PO}}_{4}^{3‐}$ (mmol m^−3^)	pH	KD (m)	PP (mg C m^−3^ day^−1^)
Celtic Sea (CS)	2011–2017	115 (83–149)	10.9 (8.9–15.8)	51 (10–140)	35.5 (35.3–35.6)	0.01 (<0.01–0.03)	0.35 (<0.01–2.25)	249 (221–272)	6.3 (1.8–8.6)	0.4 (0.0–0.5)	8.06 (8.02–8.12)	0.11 (0.07–0.16)	0.60 (0.03–3.82)
West Coast Scotland (WCS)	2012–2013	142 (115–158)	9.6 (7.3–12.6)	46 (10–140)	35.1 (34.8–35.4)	0.04 (<0.01–0.11)	0.28 (<0.01–0.55)	254 (227–274)	7.8 (3.1–12.0)	0.5 (0.2–0.8)	8.09 (8.04–8.14)	0.1 (0.09–0.16)	0.36 (0–0.86)
North Scotland (NS)	2013, 2019	127 (93–149)	8.9 (6.3–9.4)	65 (11–128)	35.1 (34.7–35.4)	0.03 (<0.01–0.09)	0.14 (<0.01–0.55)	259 (238–279)	8.1 (2.2–11.6)	0.5 (0.1–0.7)	8.08 (8.04–8.16)	0.12 (0.09–0.17)	0.24 (<0.01–0.80)
Rockall (RK)	2012–2013	222 (168–385)	9.4 (8.5–10.1)	99 (13–284)	35.3 (35.3–35.4)	0.02 (<0.01–0.07)	0.02 (0–0.25)	235 (199–262)	12.3 (9.3–13.3)	0.8 (0.6–0.9)	8.05 (8.04–8.08)	0.09 (0.07–0.11)	0.05 (0–0.37)
Faroe Shelf (FS)	2019	178 (153–207)	7.5 (6.3–9.4)	95 (10–195)	35.1 (35.1–35.2)	0.03 (<0.01–0.10)	0.04 (<0.01–0.34)	258 (237–278)	10.5 (5.2–12.1)	0.7 (0.3–0.8)	8.08 (8.06–8.15)	0.09 (0.07–0.12)	0.07 (0–0.47)
Faroe Bank (FB)	2019	115 (111–143)	8.6 (7.3–9.8)	71 (11–127)	35.2 (35.1–35.2)	0.02 (<0.01–0.07)	0.26 (<0.01–0.84)	251 (234–258)	11.0 (8.5–12.9)	0.7 (0.5–0.9)	8.08 (8.06–8.11)	0.09 (0.07–0.11)	0.40 (0–1.23)
Overall	2011–2019	115 (83–385)	10.5 (6.2–15.8)	59 (10–283)	35.4 (34.7–35.6)	0.01 (<0.01–0.10)	0.3 (<0.01–2.30)	248 (199–279)	7.4 (1.8–13.3)	0.5 (0.0–0.9)	8.06 (8.02–8.16)	0.11 (0.07–0.17)	0.50 (0–3.82)

Note

Data show bottom depth, bottom temperature (BT), mixed layer depth (MLD), salinity (Sal), current velocity (Cur), chlorophyll concentration (Chl), dissolved oxygen concentration (O_2_), nitrate concentration (${\text{NO}}_{3}^{‐}$), phosphate concentration (${\text{PO}}_{4}^{3‐}$), pH, light attenuation (KD) and primary productivity (PP).

Testing for associations between allele frequencies (two alleles for each of 6,350 loci) and 36 environmental variables (including latitude and longitude as covariates) generated a total of 457,200 tests in Samβada. After the Bonferroni correction, one allele (alternate allele at locus 100069553) was significantly associated with seven environmental variables (*p* < 0.01 for both G‐score and Wald score). These were as follows: latitude, mean current velocity, maximum pH, minimum bottom temperature, and mean, minimum and maximum salinity. This locus was also detected as one of 21 outliers under putative positive selection in BayeScan (Table 7, Figure S16). On closer inspection, we observed that the proportion of reference homozygotes at this locus was high in the Celtic Sea (96%) when compared to the other sites (33–80%). Only one homozygote for the alternate allele, a skate from West Coast Scotland, existed among all 503 genotyped skates.

**TABLE 7:** Twenty‐one SNP loci identified as potentially under positive selection in *Dipturus batis*

Locus	BayeScan	Samβada	BLASTn search		
			Description	*E*‐value	% identity
10967992	**				
10975590	*				
11001882	*				
11005420	**				
16778142	*				
16781304	**		Amblyraja radiata uncharacterized LOC116974003 (LOC116974003), ncRNA	1e‐14	88.7
			Amblyraja radiata transmembrane protein 50A (tmem50a), transcript variant X2, mRNA	2e‐13	87.3
			Amblyraja radiata beta‐1,4‐galactosyltransferase 3‐like (LOC116974216), transcript variant X1, mRNA	2e‐12	87.0
			Raja eglanteria clone 2113 Ig heavy chain (Vx, Dx1, Dx2, Jx, Cx1 and Cx2) gene region	2e‐12	87.3
			PREDICTED: Amblyraja radiata SAM and SH3 domain containing 1 (sash1), transcript variant X8, mRNA	7e‐12	85.9
16781498	**				
16782764	*		Amblyraja radiata solute carrier family 19 member 1 (slc19a1), mRNA	3e‐05	97.1
16783348	**				
16783589	**				
16783836	**				
16785038	**				
16785054	*		Danio rerio genome assembly, chromosome: 25	2e‐12	97.9
			Danio rerio strain Nadia (NA) genome assembly, chromosome: 3	2e‐12	97.9
			Zebrafish DNA sequence from clone DKEY‐106C17 in linkage group 3, complete sequence	2e‐12	97.9
			Danio rerio strain Nadia (NA) genome assembly, chromosome: 7	5e‐12	95.9
			Zebrafish DNA sequence from clone CH211‐72D16 in linkage group 17, complete sequence	5e‐12	95.9
16785163	**				
16785175	**		Amblyraja radiata uncharacterized LOC116974003 (LOC116974003), ncRNA	3e‐16	89.0
			Amblyraja radiata transmembrane protein 50A (tmem50a), transcript variant X2, mRNA	4e‐15	88.7
			Amblyraja radiata beta‐1,4‐galactosyltransferase 3‐like (LOC116974216), transcript variant X1, mRNA	5e‐14	88.4
			Raja eglanteria clone 2113 Ig heavy chain (Vx, Dx1, Dx2, Jx, Cx1 and Cx2) gene region	5e‐14	87.7
			Amblyraja radiata SAM and SH3 domain containing 1 (sash1), transcript variant X8, mRNA	2e‐13	86.3
16785304	**		Amblyraja radiata twinfilin actin binding protein 2 (twf2), transcript variant X4, mRNA	8e‐18	92.9
			Amblyraja radiata RRN3 homolog, RNA polymerase I transcription factor (rrn3), transcript variant X1, mRNA	4e‐15	90.0
			Amblyraja radiata transmembrane protein 50A (tmem50a), transcript variant X2, mRNA	5e‐14	88.6
			Amblyraja radiata beta‐1,4‐galactosyltransferase 3‐like (LOC116974216), transcript variant X1, mRNA	6e‐13	88.2
			Amblyraja radiata uncharacterized LOC116976331 (LOC116976331), ncRNA	2e‐12	87.1
100018343	*		Amblyraja radiata ATR interacting protein (atrip), transcript variant X4, mRNA	3e‐11	91.1
			Amblyraja radiata ATR interacting protein (atrip), transcript variant X3, mRNA	3e‐11	91.1
			Amblyraja radiata ATR interacting protein (atrip), transcript variant X2, mRNA	3e‐11	91.1
			Amblyraja radiata ATR interacting protein (atrip), transcript variant X1, mRNA	3e‐11	91.1
			Amblyraja radiata quinolinate phosphoribosyltransferase (qprt), transcript variant X1, mRNA	9e‐11	90.9
100018549	*				
100024701	**		Amblyraja radiata gamma‐glutamyl hydrolase‐like (LOC116981285), transcript variant X3, mRNA	1e‐08	84.1
			Amblyraja radiata gamma‐glutamyl hydrolase‐like (LOC116981285), transcript variant X2, mRNA	1e‐08	84.1
			Amblyraja radiata gamma‐glutamyl hydrolase‐like (LOC116981285), transcript variant X1, mRNA	1e‐08	84.1
			Amblyraja radiata uncharacterized LOC116978972 (LOC116978972), ncRNA	3e‐04	80.7
			Amblyraja radiata zinc finger protein 516 (znf516), transcript variant X3, misc_RNA	3e‐04	86.7
100034323	*				
100069553	**	CUR.mean** SAL.min** SAL.mean** SAL.max** Latitude** pH.max** BT.min**			

Note

Significance at *p* < 0.05 (*) and *p* < 0.01 (**) is shown for outlier detection in BayeScan. Loci associated with environmental variables in multiple logistic regression using Samβada following the Bonferroni correction at *p* < 0.05 (*) and *p* < 0.01 (**) are also shown. Abbreviations refer to salinity (SAL) and bottom temperature (BT). The top BLASTn hits from NCBI with an *E*‐value < 0.01 for each locus are reported.

The blastn search produced ambiguous hits for seven out of 21 outlier loci (Table 7). Percentage sequence identity ranged from 80 to 97%. The majority of the hits were against mRNA and ncRNA transcript variants for *Amblyraja radiata*, but there were also matches against the immunoglobulin heavy chain gene region for *Raja eglanteria*.

## 4 DISCUSSION

The objectives of this study were to characterize the contemporary population structure, estimate effective population sizes and investigate putative patterns of adaptation along environmental gradients in the critically endangered blue skate *Dipturus batis*. We identified a clear genetic discontinuity across the Rockall Trough contrasting high gene flow along the British continental shelf. The results corroborate the findings of Frost et al. (2017), who demonstrated this using microsatellite markers on a subset of the samples used in this study. With additional samples from these and Faroese sites, we identified another genetic discontinuity between the Faroe Islands and the British shelf, though the evidence for this discontinuity was not unequivocal. Effective population size estimates were relatively high in the Celtic Sea and Rockall, but sufficiently low in Scotland and the Faroe Islands to be considered a potential conservation concern. We also identified 21 candidate SNPs under selection, including one associated with environmental variables that are expected to shift in response to a changing climate, which may have implications for the future realized niche of *D. batis*.

The isolation of offshore populations of *D. batis*, which contrast the high level of coastal connectivity, is a recurring pattern for coastal elasmobranchs (Le Port & Lavery, 2012) that may be driven by the presence of bathymetric barriers. The Rockall Bank was the most genetically isolated population in *D. batis*, occurring on an offshore plateau ~250 km from the British continental shelf and surrounded by deep (>1,000 m) waters that exceed the species’ reported depth range. Similarly, the Faroe Shelf and Bank are separated from the Scottish shelf by the Rockall Trough to the south and the ~1,200‐m‐deep Faroe–Shetland Channel to the east. Several studies have attributed the genetic isolation of Rockall and Faroe Island populations of benthic and benthopelagic fishes (Gonzalez et al., 2014; Johansen et al., 2020; Mattiangeli et al., 2002; Régnier et al., 2017; Saha et al., 2015) and squid (Shaw et al., 1999) to these bathymetric barriers. Unlike these animals, skates are almost exclusively epibenthic throughout their life cycle, laying their egg cases (mermaid's purses) on the sea bed (Last et al., 2016) and spending the majority of their life near the sea floor (Wearmouth & Sims, 2009). Therefore, the bathymetric barriers are unlikely to be readily overcome unless skates swim long distances high in the water column.

The patterns of population structure observed in *D. batis* are also likely influenced by the species’ highly resident behaviour. The proximity of close relatives identified in this study is indicative of site‐attached behaviour, and supports the initial results of tagging in the Celtic Sea, which demonstrates that *D. batis* almost exclusively remain within relatively confined shallow areas (<200 m) of the continental shelf (Bendall et al., 2018). Nonetheless, the results indicate high levels of gene flow spanning over 1,000 km along the British continental shelf from the Celtic Sea to northern Scotland. Tagging experiments investigating the related and more extensively studied flapper skate *D. intermedius* along the Scottish coast may provide inference about movement patterns in *D. batis*. Despite high levels of residency (Little, 1998; Wearmouth & Sims, 2009), around 25% of *D. intermedius* are vagrant or ‘transient’ individuals (Neat et al., 2015) and have been recaptured up to 900 km from their release sites (Little, 1998). In contrast, the extent of *D. batis* movement appears to be more restricted (early results from Bendall et al. (2018) indicated a maximum recapture distance of 170 km), but the occurrence of transient individuals is conceivable. If the scarcity of samples from Scotland and their low effective population sizes are indicative of low abundances in this area, the occurrence of occasional breeding migrants from larger populations such as the Celtic Sea could explain the genomic homogeneity observed. It could be argued that the Scottish samples in our study were themselves transient individuals from the Celtic Sea; however, these samples were predominantly juveniles (21–108 cm, Table 1) and are unlikely to have undertaken long‐distance movements. In fact, individuals in the immature size range were collected at all sites (length at first maturity <115 cm for males, <125 cm for females; McCully et al., 2012), indicative of restricted resident populations utilizing nursery habitats at or near each of the sample sites.

Abundance estimates are vital to establish effective conservation strategies but are currently lacking for *D. batis*. Our pooled effective population size (*N*_e_) estimates indicated that the British Shelf and Rockall had the highest *N*_e_ (~21,000 and ~11,000 individuals, respectively), though the former were dominated by high population densities in the Celtic Sea. The Faroe Islands had a much lower *N*_e_ (~2,300 individuals). The minimum *N*_e_ required to maintain the evolutionary potential of a population has been a subject of debate, with proposed conservation thresholds set between 500 and 5,000 individuals (reviewed in Allendorf et al., 2013), below which populations may be prone to the loss of genetic diversity, higher inbreeding and the accumulation of deleterious mutations. Whereas the Celtic Sea had relatively high *N*_e_, the estimates for the Scottish, Rockall and Faroese populations could be construed as being of conservation concern. We note that *N*_e_ estimation from high‐throughput sequencing data can be subject to bias due to violations to the assumptions of unlinked loci that are difficult to correct for without knowledge of the species’ genomic architecture (Waples et al., 2016). Its interpretation also depends on the life history and demography of the species and should ideally be compared with census population size (*N*_c_) (Lieber et al., 2020; Waples et al., 2018). Although these factors may affect the reliability of our *N*_e_ estimates, they provide an important first estimate of *D. batis* abundance across its distribution range and serve as initial indicators of their conservation status. The identification of close relatives among our samples suggests that *N*_c_ could be estimated using close‐kin mark–recapture, provided an appropriate survey design (Bravington et al., 2016; Bravington et al., 2016).

The seascape genomics analysis revealed a significant association between one locus under putative positive selection (which was identified as an outlier in BayeScan; Table 7) and a number of abiotic variables expected to be influenced by climate change. Previous work has demonstrated that *D. batis* may be tolerant to a narrower thermal range than its parapatric congener, *D. intermedius*, where their ranges overlap (Frost et al., 2020); this may lead to differential shifts in fitness under projected climate change. Not only did our findings identify an association between a putatively adaptive locus and minimum bottom temperature, but also associated with pH, salinity and current velocity. A dominance of homozygotes for the reference allele was found in the Celtic Sea (96% of individuals, compared with 33–80% of individuals elsewhere), where bottom waters are warmer, more saline and more acidic, and have slower mean current velocities than the northern sites. The pronounced geographic pattern at this locus occurs despite apparently high levels gene flow between Scottish sites and the Celtic Sea, suggesting that a strong selective force may be acting on this locus or on a closely linked one.

Climate change is expected to lead to warmer, fresher and more acidic waters around the British Isles by the end of the century, with an increasing frequency of extreme oceanographic events (MCCIP, 2020). These three abiotic variables are likely to have important biological consequences for a benthic elasmobranch. Osmoregulation and acid–base regulation are vital physiological functions for the maintenance of homeostasis in the marine environment. The implications of these changes on *D. batis* remain unclear. The closest comparison can be drawn from experimental work on little skate *Leucoraja erinacea* from the western North Atlantic Ocean, in which individuals from northern parts of the range (colder and less thermally variable) were more sensitive to acidification and warming, as indicated by a lower aerobic capacity and slower recovery following anaerobic activity (Di Santo, 2016). Other studies have demonstrated that warming and acidification could impact elasmobranch hunting ability through impaired olfaction and increased energetic demands (Pistevos et al., 2015), influence juvenile skeletal development (Di Santo, 2019) and lead to denticle corrosion (Dziergwa et al., 2019). The association suggested with mean current velocity could relate to gas exchange rates during respiration when resting on the sea floor, or to the energetic costs of swimming. Due to a lack of reference genomes for *D. batis* and its relatives, BLAST searches for this and the 20 other putatively adaptive loci did not produce any unambiguous hits. Whereas this locus could be part of a functional sequence, it could also be physically linked to a gene under selection. Future investigations will benefit from more targeted approaches as genomic resources for elasmobranchs improve.

Our results provide an updated assessment of *D. batis*’ population structure, regional abundance and movement patterns that will have immediate relevance for the conservation of this critically endangered species, specifically through the delimitation of current and future spatial management units. In view of our findings suggesting that climate change may affect populations of *D. batis* differentially across their distributional range, it may be necessary to consider the long‐term efficacy of any conservation strategies. The potential impacts of recent and projected oceanographic changes in the North‐East Atlantic Ocean (MCCIP, 2020), which include unprecedented freshening events (Penny Holliday et al., 2020), are important to consider for populations already fragmented by fishing activity. The seascape genomics results not only provide further evidence of the role of temperature in determining skate distributions and connectivity in the North‐East Atlantic Ocean (Frost et al., 2020; Griffiths et al., 2010), but also suggest a potential influence of other environmental variables such as salinity and pH. Should environmental changes incur a physiological cost to which site‐attached coastal elasmobranchs such as *D. batis* cannot adapt, a poleward distribution shift or a move to greater depths as observed in other species (Barton et al., 2016; Brattegard, 2011; Chaudhary et al., 2021; Perry et al., 2005) could constrain their habitat to the fringes of the continental shelf, or lead to further population fragmentation. While some studies have documented the physiological consequences that warming and acidification can have on elasmobranchs (Di Santo, 2016, 2019; Dziergwa et al., 2019; Pistevos et al., 2015), our results suggest a possible genetic basis for adaptation to such environmental changes. Future studies benefiting from improved genomic resources for *D. batis* may address this hypothesis in greater detail. Nonetheless, our study demonstrates the efficacy of using increasingly affordable genome‐wide approaches to address fundamental knowledge gaps for one of many threatened and data‐deficient elasmobranchs, leading to better spatial management.

## CONFLICT OF INTEREST

The authors declare no conflicts of interest.

## Supporting information

Supplementary Material

## Data Availability

The data that support the findings of this study are openly available in the Dryad Digital Repository http://doi.org/10.5061/dryad.98sf7m0kc
