# Population and seascape genomics of a critically endangered benthic elasmobranch, the blue skate *Dipturus batis*

**DOI:** 10.1111/eva.13362

**Published:** 2023-02-13

This paper is a part of the Special Issue ‘A decade of progress in Marine Evolutionary Biology’ but was published in error in the normal run of the journal, and the paper can be found at this link: https://onlinelibrary.wiley.com/doi/epdf/10.1111/eva.13327

The publisher apologizes for the error.
