# Supplementary material for: Population and seascape genomics of a critically endangered benthic elasmobranch, the blue skate Dipturus batis
Source: Evol Appl. 2021 Dec 7;15(1):78–94. doi: 10.1111/eva.13327 (PMC8792474; doi:10.1111/eva.13327)
Supplement: Supplementary file 1 — Supplementary Material [file EVA-15-78-s001.docx]

**Supplementary material**

i) Further details of sample provenance from the Celtic Sea

Samples of blue skate from the Celtic Sea were obtained during a fishery-dependent common skate survey performed in collaboration with the fishing industry in 2011, and from 2014-2017. Surveys were run during the late summer to autumn (August-October). Fixed trammel nets were deployed along a transect of stations running 12-80 nm to the south and west of Newlyn, Cornwall, UK, with additional exploratory stations outside the main transect area also surveyed (Supplementary figure 1). Further details of the sampling protocol are described in Bendall et al. (2018).


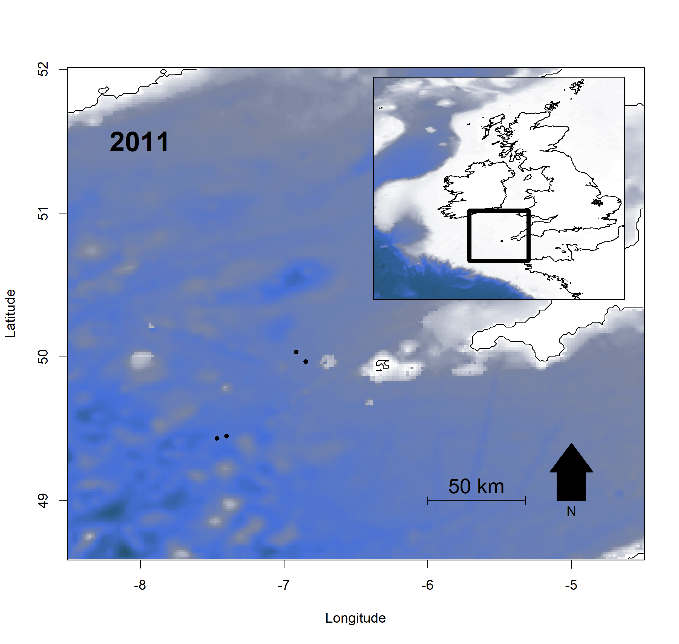

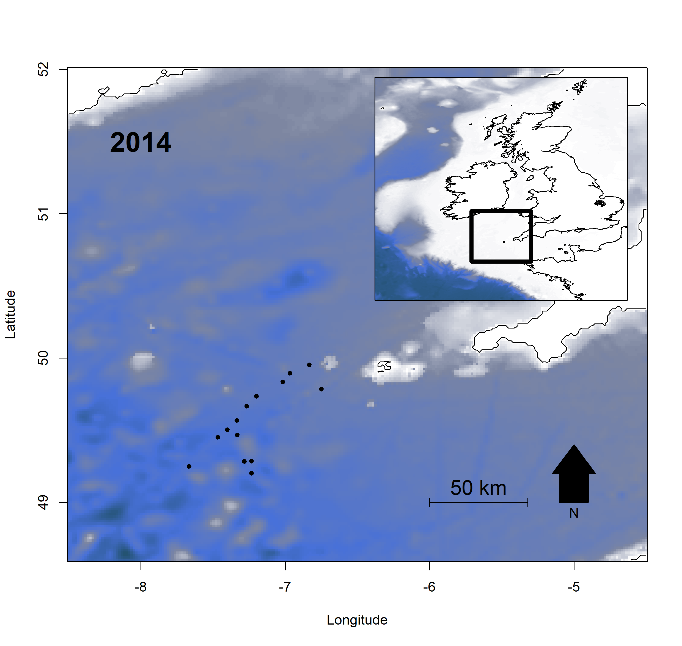

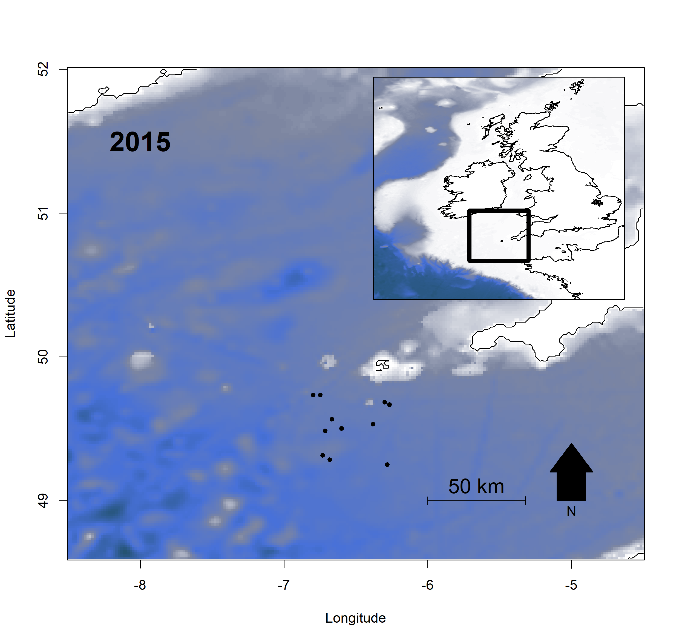

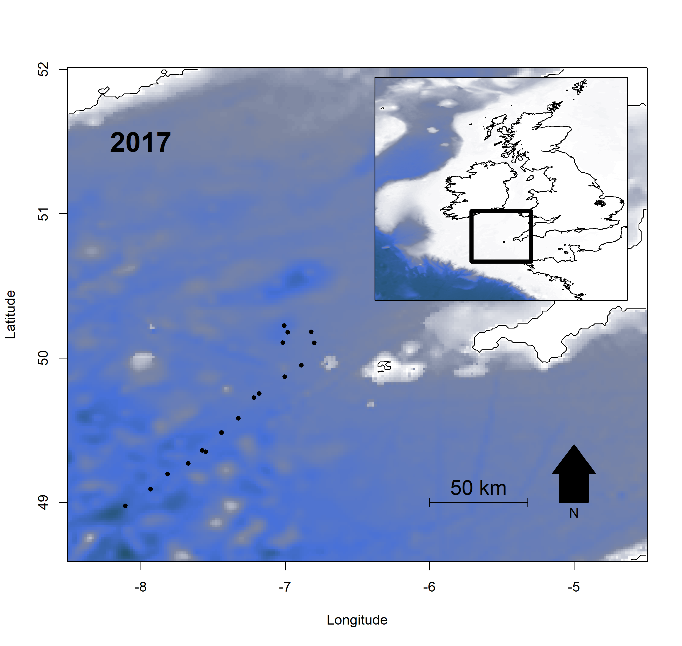


Supplementary figure 1: Trammel net haul locations where blue skate (*Dipturus batis*) samples were collected in 2011, 2014, 2015, and 2017 in the Celtic Sea.

ii) Filtering of the SNP dataset.

| Supplementary table 1: Summary of filtering steps for single nucleotide polymorphism (SNP) data obtained using DArTseq on blue skate *Dipturus batis* | | | |
| --- | --- | --- | --- |
| **Filtering step** | **No. loci removed** | **No. loci remaining** |  |
| Raw data |  | 17,621 |  |
| Call rate <80% | 3,329 | 14,292 |  |
| Duplicate loci | 1,352 | 12,940 |  |
| Monomorphic loci | 77 | 12,863 |  |
| Minor Allele Frequency (MAF) < 0.05 | 5,800 | 7,063 |  |
| Loci out of Hardy-Weinberg proportions in at least 2 populations | 521 | 6,542 |  |
| Loci in linkage disequilibrium | 192 | 6,350 |  |

Supplementary table 2: Results of exact test for conformation of loci to Hardy-Weinberg proportions, based on Monte Carlo permutation of alleles (Guo and Thompson 1992) with 1000 replicates, implemented using the R package *pegas*. Number of loci out of Hardy-Weinberg are shown before and after Benjamini and Hochberg (1995) false-discovery rate (FDR) correction. Site names are abbreviated for the Celtic Sea (CS), West Coast Scotland (WCS), Northern Scotland (NS), Rockall (RK), the Faroe Bank (FB), and the Faroe Shelf (FS).

| **Sampling location** | **No. of tests involved** | **No. of loci out of HW** | **No. loci out of HW after FDR correction** |
| --- | --- | --- | --- |
| CS | 42,378 | 2,170 | 1,509 |
| WCS | 42,378 | 516 | 63 |
| NS | 42,378 | 128 | 3 |
| RK | 42,378 | 1,323 | 610 |
| FB | 42,378 | 99 | 3 |
| FS | 42,378 | 197 | 6 |
| All samples | 7,063 | 2,528 | 2,092 |

No. of loci out of HWE in at least 1 sampling location: 1,640
No. of loci out of HWE in at least 2 sampling locations: 521
No. of loci out of HWE in at least 3 sampling locations: 33
No. loci out of HWE in at least 4 sampling locations: 0

iii) The power of relationship inference based on 6,350 SNP loci, and decision making for log-likelihood thresholds in relationship tests. A Monte Carlo simulation approach was implemented using *CKMRsim* (Anderson, https://doi.org/10.5281/zenodo.820162), simulating 10,000 relationship pairs (parent-offspring PO, full-sibling FS, half-sibling HS, and unrelated U) based on observed allele frequencies in our genotype data from 503 *Dipturus batis* individuals.

Supplementary table 3: False positive rates (FPR) and associated log-likelihood (logl) thresholds in each of six pairwise relationship tests (involving parent-offspring PO, full-sibling FS, half-sibling HS, and unrelated U pairs) based on simulated data, at false-negative rates of 0.001.

| **Test** | **FPR** | **logl threshold** |
| --- | --- | --- |
| PO/U | 2.23x10^-294^ | 667 |
| FS/U | 7.02x10^-268^ | 606 |
| HS/U | 5.62x10^-56^ | 118 |
| PO/FS | 3.94x10^-286^ | 95 |
| PO/HS | 2.87x10^-289^ | 192 |
| FS/HS | 4.53x10^-255^ | 164 |


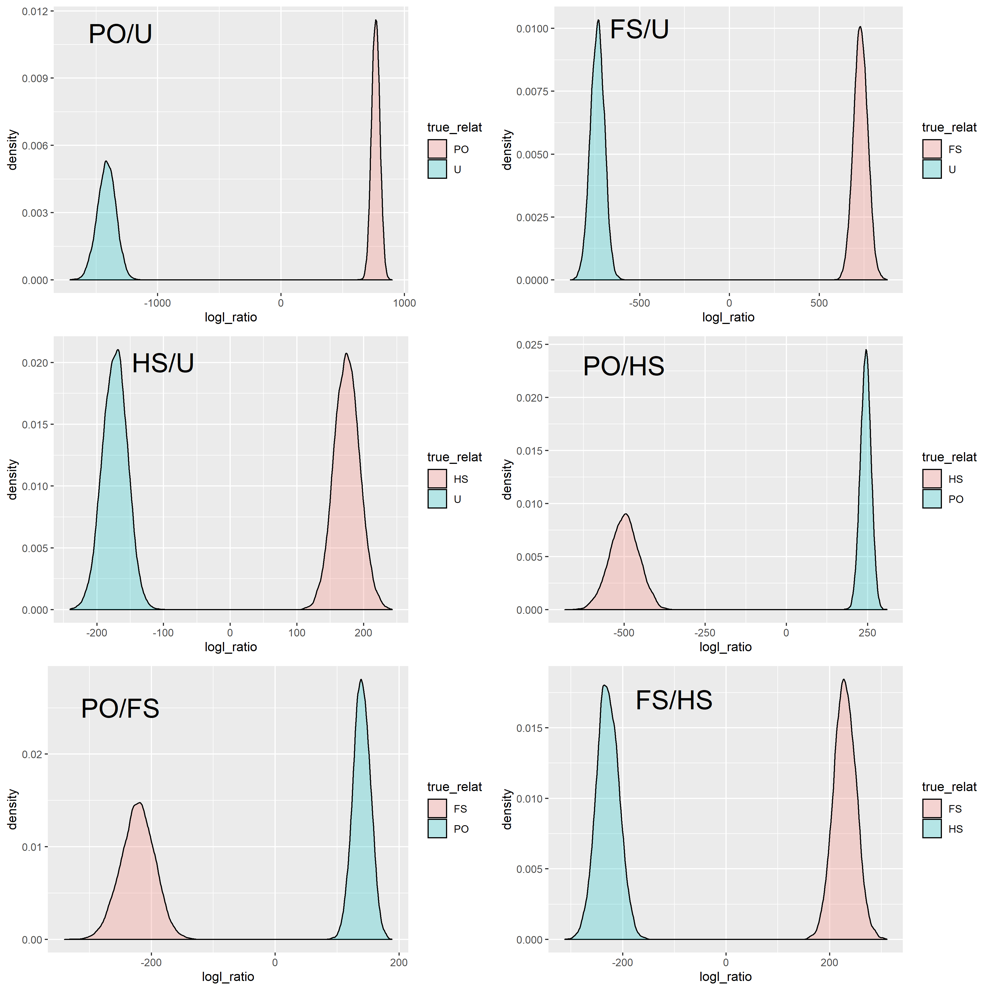


Supplementary figure 2: Density plots depicting the log-likelihood ratios of simulated relationship pairs in each pairwise test involving parent-offspring (PO), full-sibling (FS), half-sibling (HS), and unrelated (U) pairs. The distributions were used by CKMRsim to calculate false-positive and false-negative rates associated with each log-likelihood ratio.

iv) Preliminary analysis of population structure among 493 *D. batis* from six sampling locations using fastSTRUCTURE (Raj et al., 2014). The analysis was performed to justify the sub-sampling of individuals from different populations for the full STRUCTURE analysis, ensuring that no structure within sampling locations would go undetected.


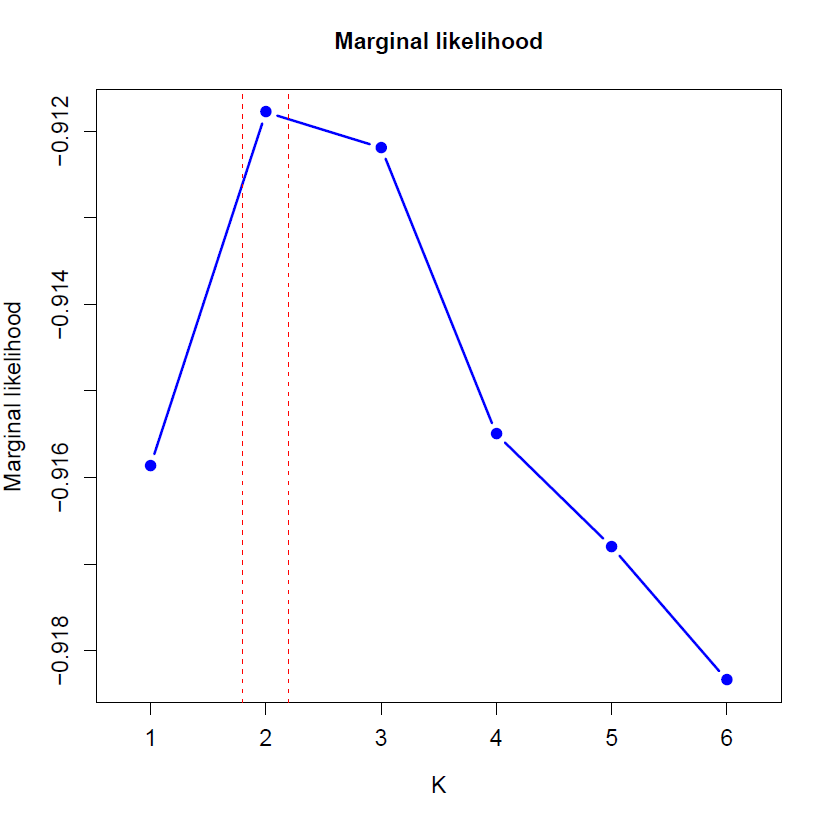


Supplementary figure 3: Marginal likelihood of different values of prior sub-population number (K, ranging from K=1 to K=6) following analysis of 493 *Dipturus batis* from six North-East Atlantic sampling locations. The figure was generated using StructureSelector (Li & Liu, 2018).


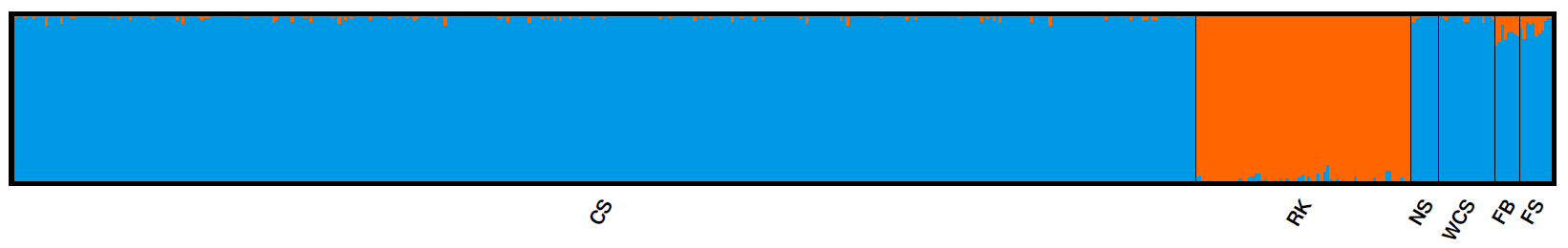


Supplementary figure 4: Summary of clustering in fastSTRUCTURE analysis for K=2, for 493 *D. batis* samples collected from the Celtic Sea (CS), Rockall (RK), North Scotland (NS), West Coast Scotland (WCS), the Faroe Bank (FB) and the Faroe Shelf (FS). The figure was generated in CLUMPAK (Kopelman et al., 2015), implemented through StructureSelector (Li & Liu, 2018).

v) Exploratory analysis of population structure for 379 *D. batis* sampled in the Celtic Sea between 2011 and 2017. Analysis was performed using the Bayesian clustering algorithm implemented in STRUCTURE (Pritchard et al., 2000), and results interpretation using STRUCTURE Harvester (Earl & VonHoldt, 2002) and CLUMPAK (Kopelman et al., 2015). We used an admixture model with correlated allele frequencies, a burn-in length of 100,000 followed by 50,000 MCMC runs, and performed 3 iterations for each prior sub-population number K (ranging from K=1 to K=5).


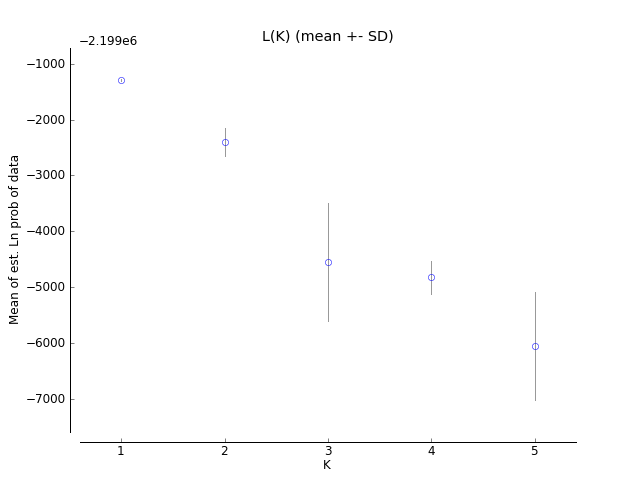

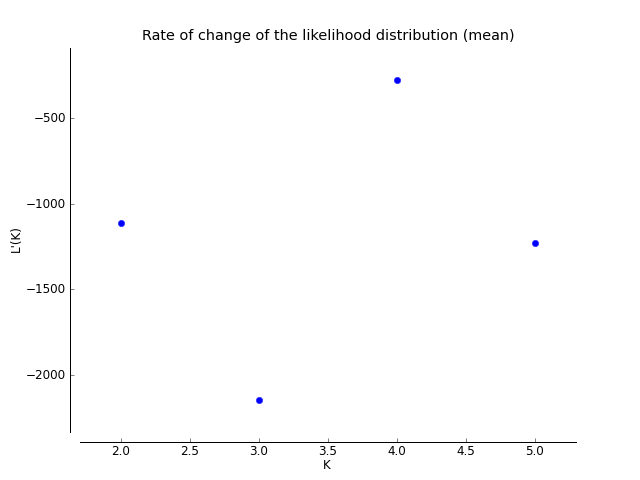


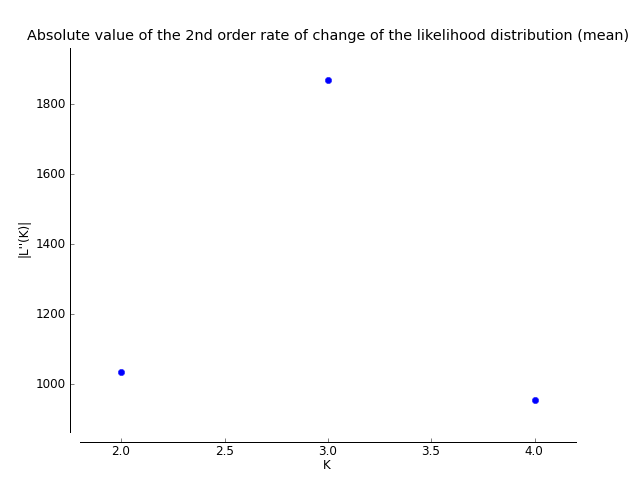

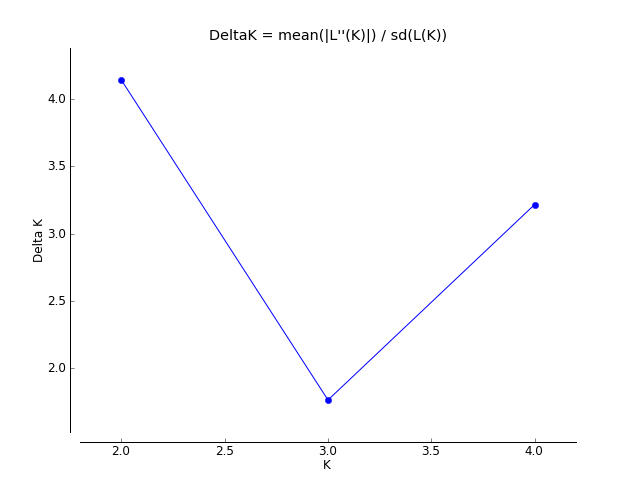


Supplementary figure 5: Interpreting the results of STRUCTURE runs on 379 Celtic Sea D. batis. The likelihood of the data and various transformations to calculate delta-K using the Evanno et al. (2005) method implemented in Structure Harvester (Earl and vonHoldt, 2012) are shown.

K=2

K=3


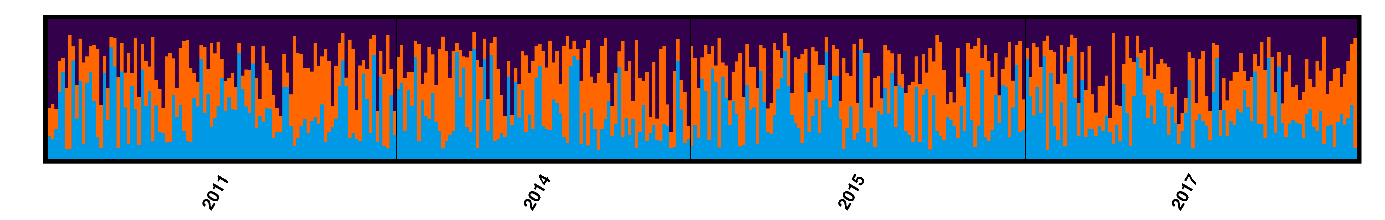


K=4

K=5

Supplementary figure 6: Summary of clustering for K=2 through K=5 generated in CLUMPAK (Kopelman et al. 2015), for 379 D. batis samples collected from the Celtic Sea in 2011, 2014, 2015 and 2017.

vi) Final analysis of population structure for 57 *D. batis* from the study’s entire geographic range. Analysis was performed using the Bayesian clustering algorithm implemented in STRUCTURE (Pritchard et al., 2000), and results interpretation using STRUCTURE Harvester (Earl & VonHoldt, 2002) and CLUMPAK (Kopelman et al., 2015). We used an admixture model with correlated allele frequencies, a burn-in length of 300,000 followed by 500,000 MCMC runs, and performed 5 iterations for each prior sub-population number K (ranging from K=1 to K=6).


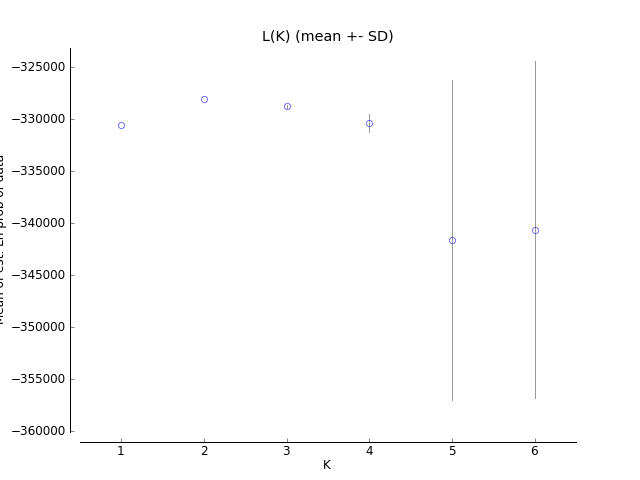

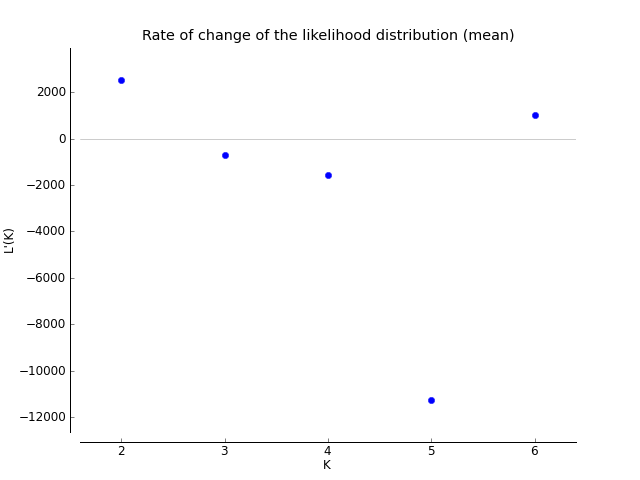


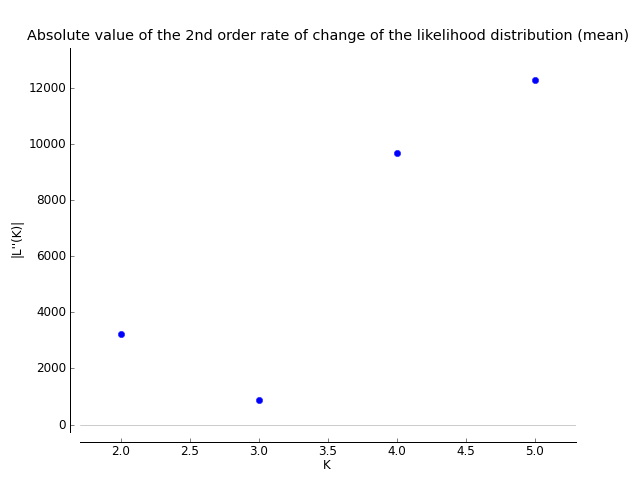

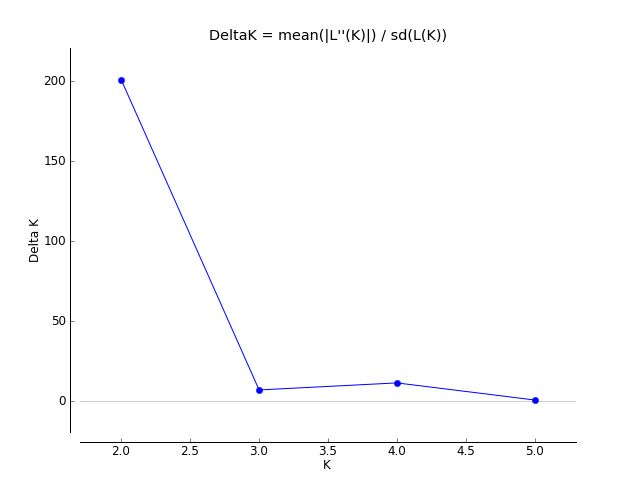


Supplementary figure 7: The likelihood of the data and various transformations to calculate delta-K using the Evanno et al. (2005) method implemented in Structure Harvester (Earl and vonHoldt, 2012).

K=2

K=3

K=4

K=5

K=6

Supplementary figure 8: Summary of clustering for K=2 through K=6 generated in CLUMPAK (Kopelman et al. 2015) for 57 *Dipturus batis* samples collected from the Celtic Sea (CS), West Coast Scotland (WCS), North Scotland (NS), Rockall (RK), the Faroe Shelf (FS), and the Faroe Bank (FB). Each individual is represented by a vertical line with the proportion of assignment to a cluster indicated by 2 to 6 colours.

vii) Analysis details, and further analyses, using discriminant analysis of principal components (DAPC).


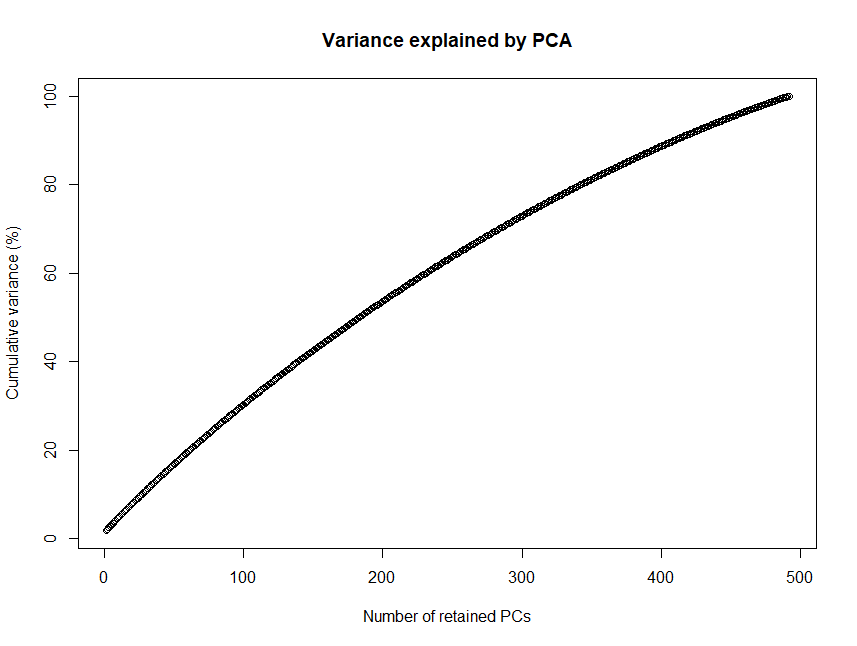

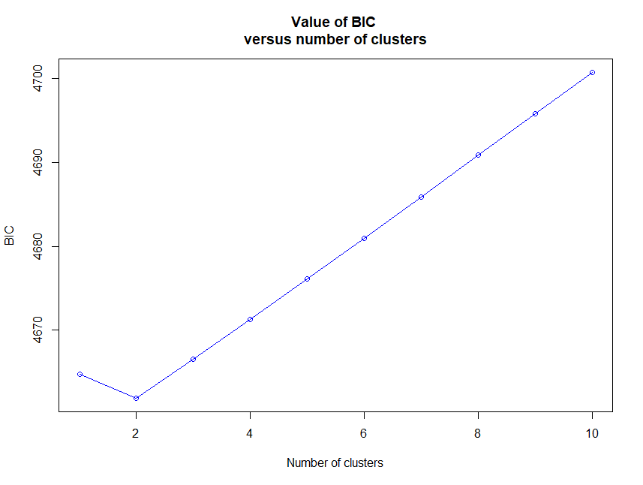


Supplementary figure 9: Analysis plots for a discriminant analysis of principal components (DAPC) on 6,350 SNPs from 493 individuals of Dipturus batis, using the find.clusters function implemented in the R package adegenet. The figures depict the cumulative variance (%) explained by 493 principal components, and the Bayesian Information Criterion (BIC) for each inferred number of clusters ranging from 1 to 10.


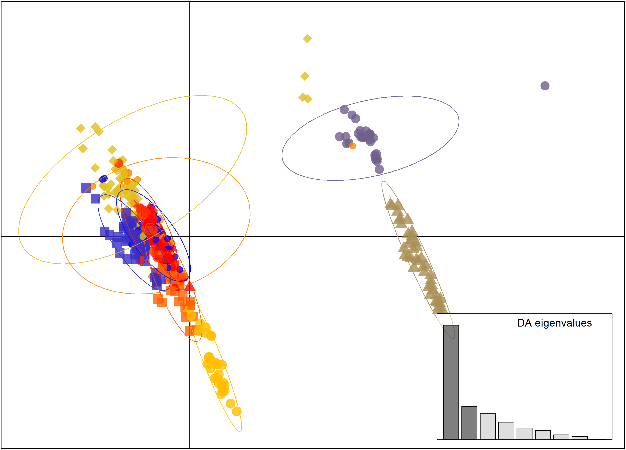

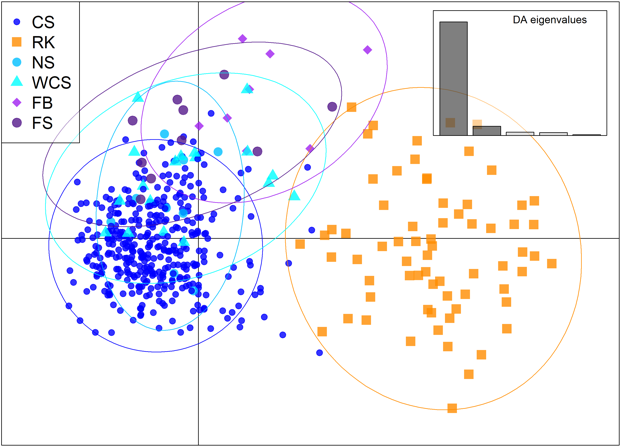


Supplementary figure 10: Discriminant analysis of principal components (DAPC) depicting the diversity between 493 blue skates in 10 inferred groups (left) and 6 groups defined by geographical location (right), using only 21 outlier loci. DAPC results were plotted using all 21 PCs (explaining 100% of variance), after retaining 9 and 5 discriminant functions of inferred and pre-defined groups respectively. 95% inertia ellipses are shown. Site names are abbreviated for the Celtic Sea (CS), West Coast Scotland (WCS), Northern Scotland (NS), Rockall (RK), the Faroe Bank (FB), and the Faroe Shelf (FS).


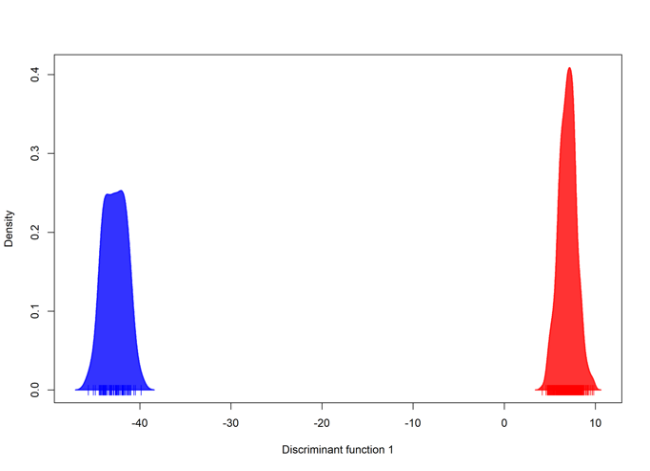

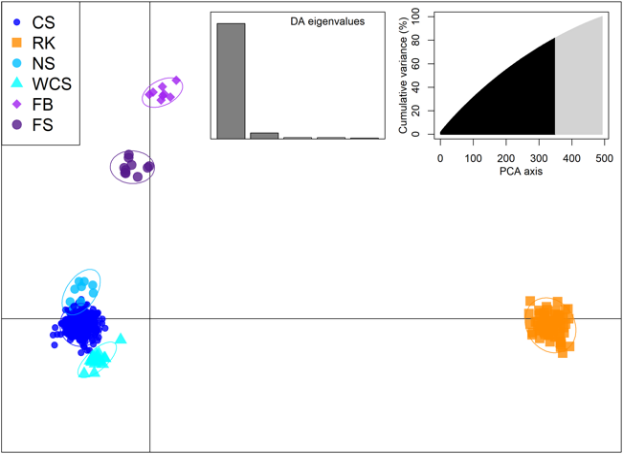


Supplementary figure 11: Discriminant analysis of principal components (DAPC) depicting the diversity between 493 blue skates in 2 inferred groups (left) and 6 groups defined by geographical location (right), using only 6,329 neutral loci, after removal of 21 putative outlier loci. The plots were produced after retaining 350 PCs (82% of variance) and 1 and 5 discriminant functions for inferred and pre-defined groupings respectively. 95% inertia ellipses are shown. Site names are abbreviated for the Celtic Sea (CS), West Coast Scotland (WCS), Northern Scotland (NS), Rockall (RK), the Faroe Bank (FB), and the Faroe Shelf (FS).

viii) Results of principal component analysis (PCA) of 503 individuals of *Dipturus batis* genotyped across a subset of 3,540 SNPs, using the R function *prcomp*.


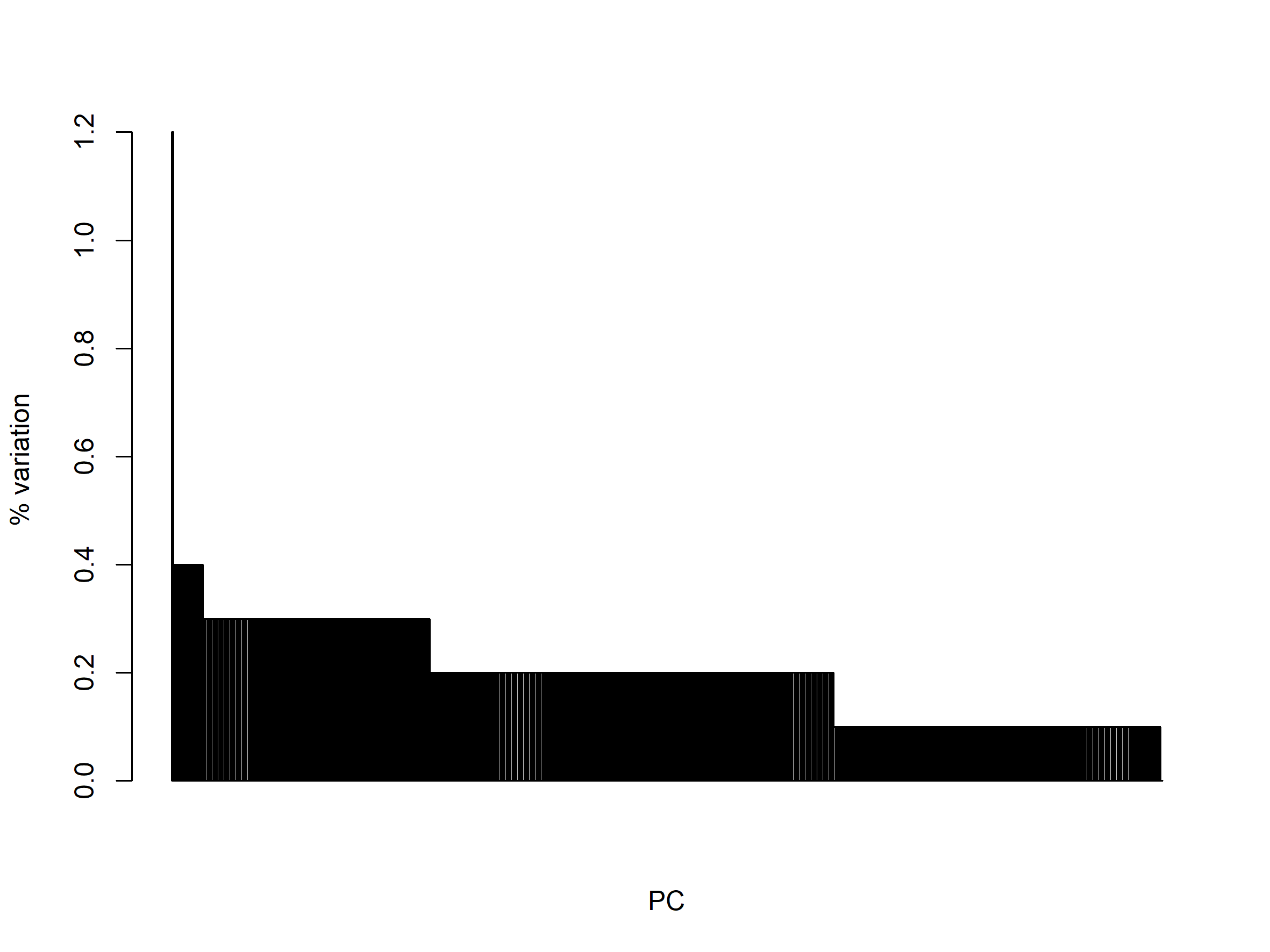


Supplementary figure 12: Percentage of variation explained by each of 503 principal components


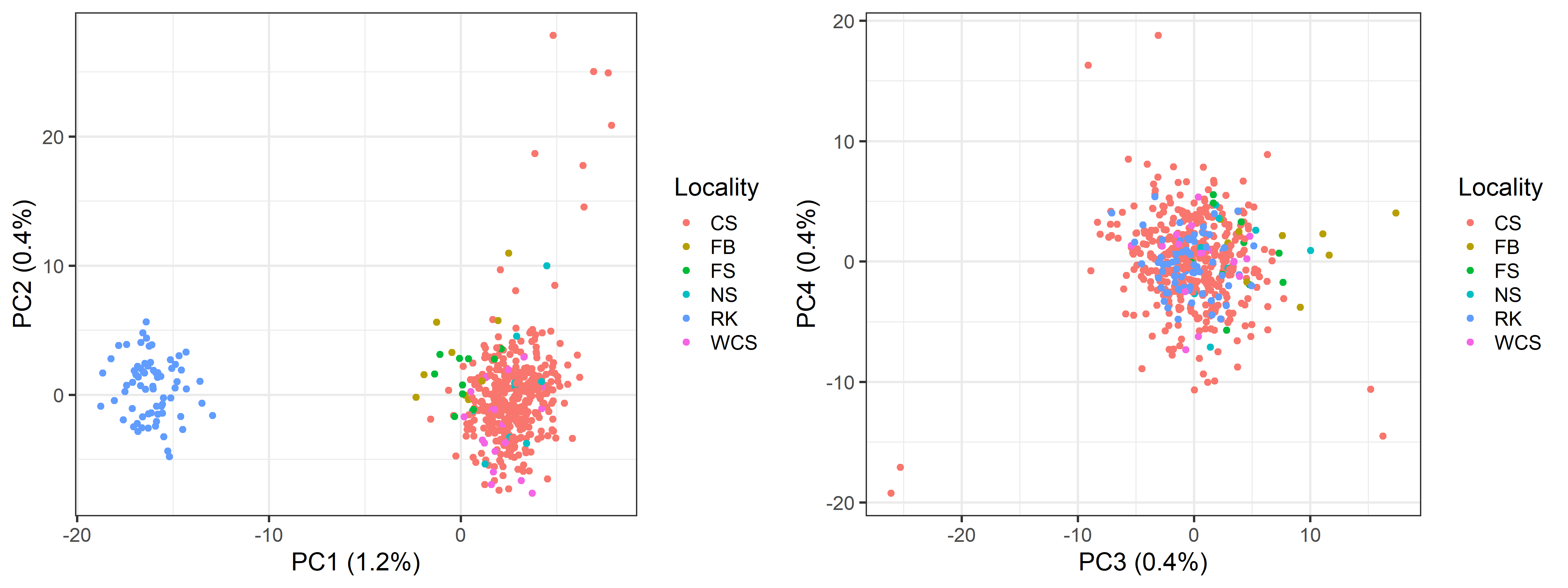


Supplementary figure 13: Principal component analysis of 503 D. batis from 6 sampling locations across 3,540 SNP loci, showing variation across the first 4 principal components. Site names are abbreviated for the Celtic Sea (CS), West Coast Scotland (WCS), Northern Scotland (NS), Rockall (RK), the Faroe Bank (FB), and the Faroe Shelf (FS).

ix) Estimates of effective population size (*N_e_*) for *Dipturus batis* at individual sampling sites

Supplementary table 4: *N_e_* for *D. batis* at 6 sampling locations, estimated using the linkage-disequilibrium method in NeEstimator. Estimates are shown for three critical values (Crit = 0.05, 0.02, and 0.01), and 95% confidence intervals by Jackknifing over individuals are shown in parentheses. Sample sizes (N) are also shown. Site names are abbreviated for the Celtic Sea (CS), West Coast Scotland (WCS), Northern Scotland (NS), Rockall (RK), the Faroe Bank (FB), and the Faroe Shelf (FS).

| Sample site | N | Crit=0.05 | Crit=0.02 | Crit=0.01 |
| --- | --- | --- | --- | --- |
| CS | 379 | 20,887  (17,312-26,307) | 21,011  (17,550-26,161) | 21,001  (17,551-26,127) |
| RK | 69 | 11,299  (3,903-∞) | 14,475  (3,810-∞) | 18,983  (3,943-∞) |
| NS | 9 | ∞ | ∞ | ∞ |
| WCS | 18 | 5,526  (328-∞) | ∞  (330-∞) | ∞  (330-∞) |
| FB | 8 | ∞ | ∞ | ∞ |
| FS | 10 | ∞ | ∞ | ∞ |
| All | 493 | 1,137  (880-1,572) | 1,146  (894-1,566) | 1,146  (894-1,566) |

x) Environmental characterisation and seascape genomics analysis


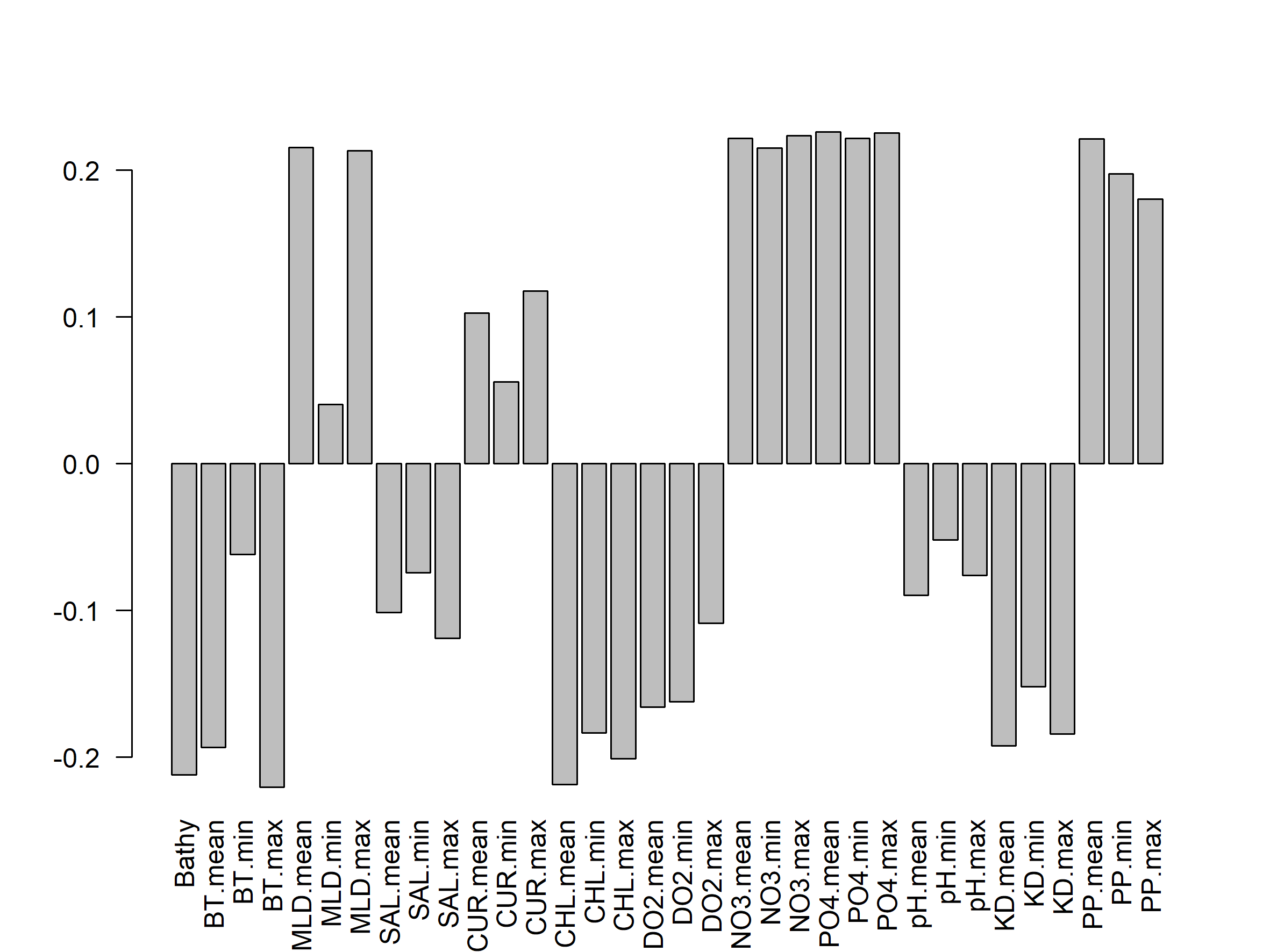

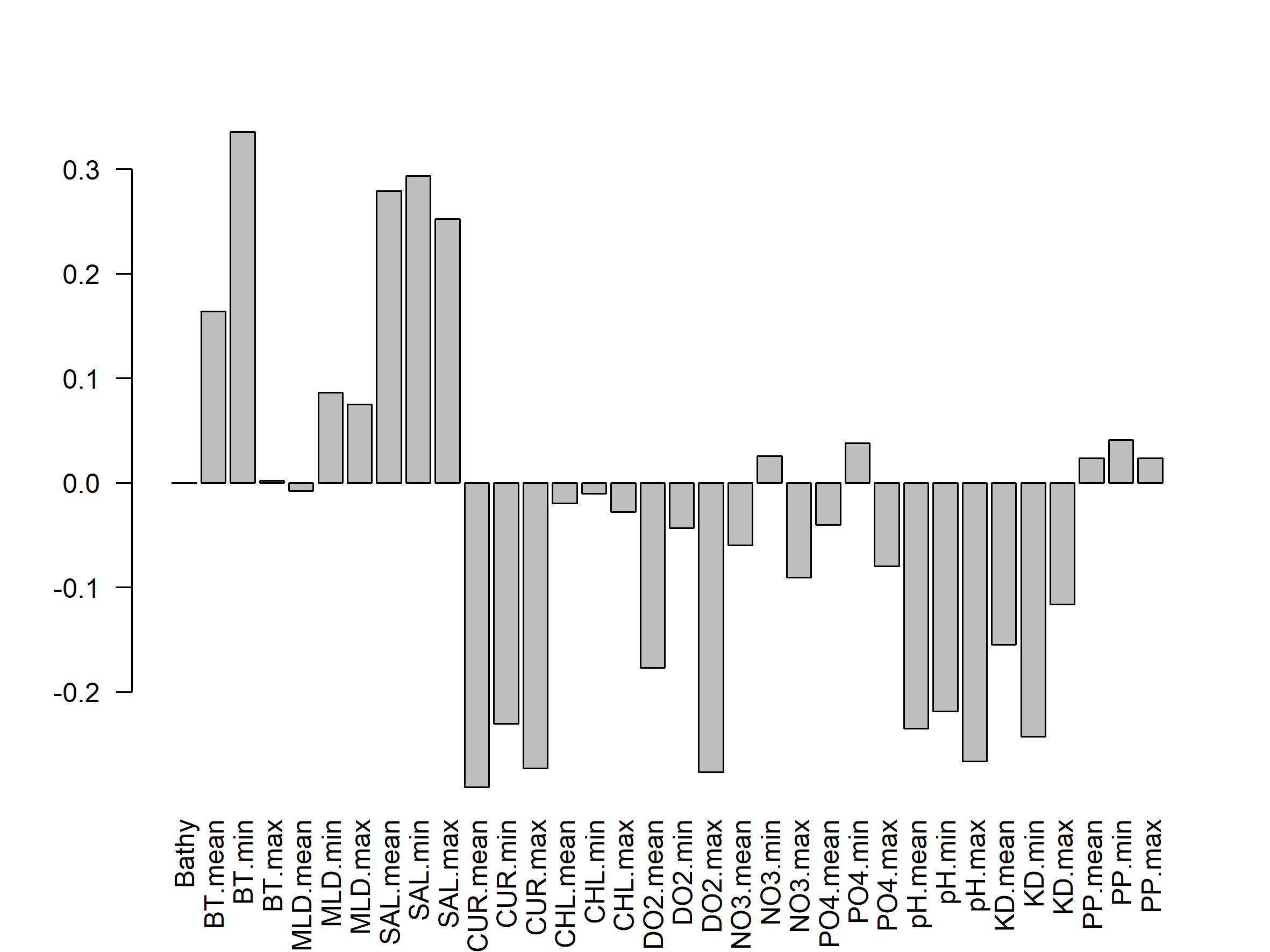


Supplementary figure 14: PCA rotation values, depicting the relative contribution of each environmental variable to PC1 (left) and PC2 (right). Variable names are abbreviated for bottom depth (bathy), bottom temperature (BT), mixed layer depth (MLD), salinity (SAL), current velocity (CUR), chlorophyll concentration (CHL), dissolved oxygen concentration (DO2), nitrate concentration (NO3), phosphate concentration (PO4), pH, light attenuation (KD), and primary productivity (PP).


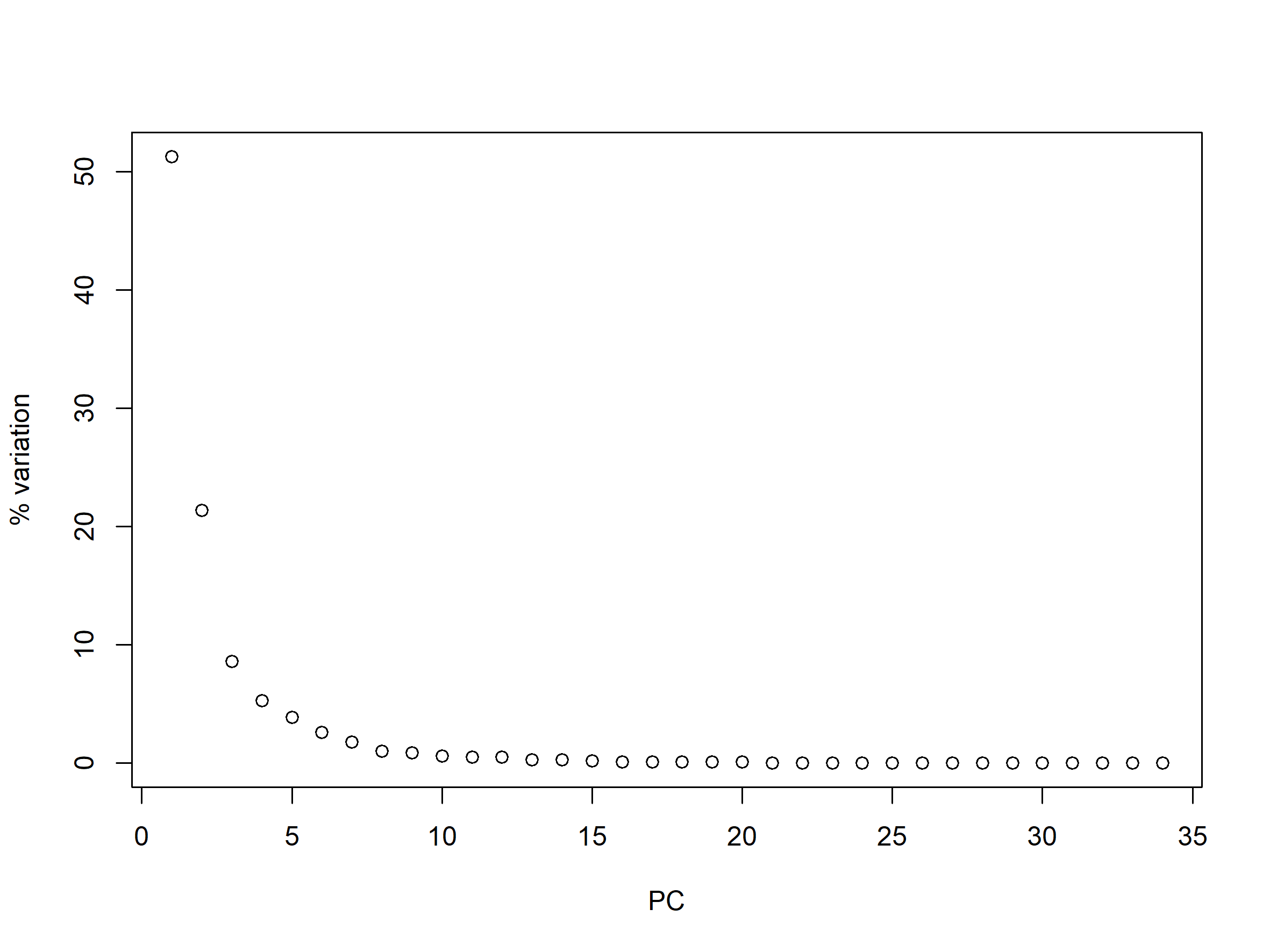


Supplementary figure 15: Percentage variation among sites per principal component in a PCA with 34 environmental variables.

Supplementary table 5: The number of significant allele-environment tests before and after Bonferroni correction for P-values derived from G and Wald scores.

| Criteria | Gscore | Waldscore |
| --- | --- | --- |
| All tests | 457,200 | 457,200 |
| P<0.05 | 28,143 | 25,820 |
| P<0.01 | 6,683 | 5,757 |
| Bonferroni P<0.05 | 8 | 7 |
| Bonferroni P<0.01 | 8 | 7 |

xi) Outlier loci


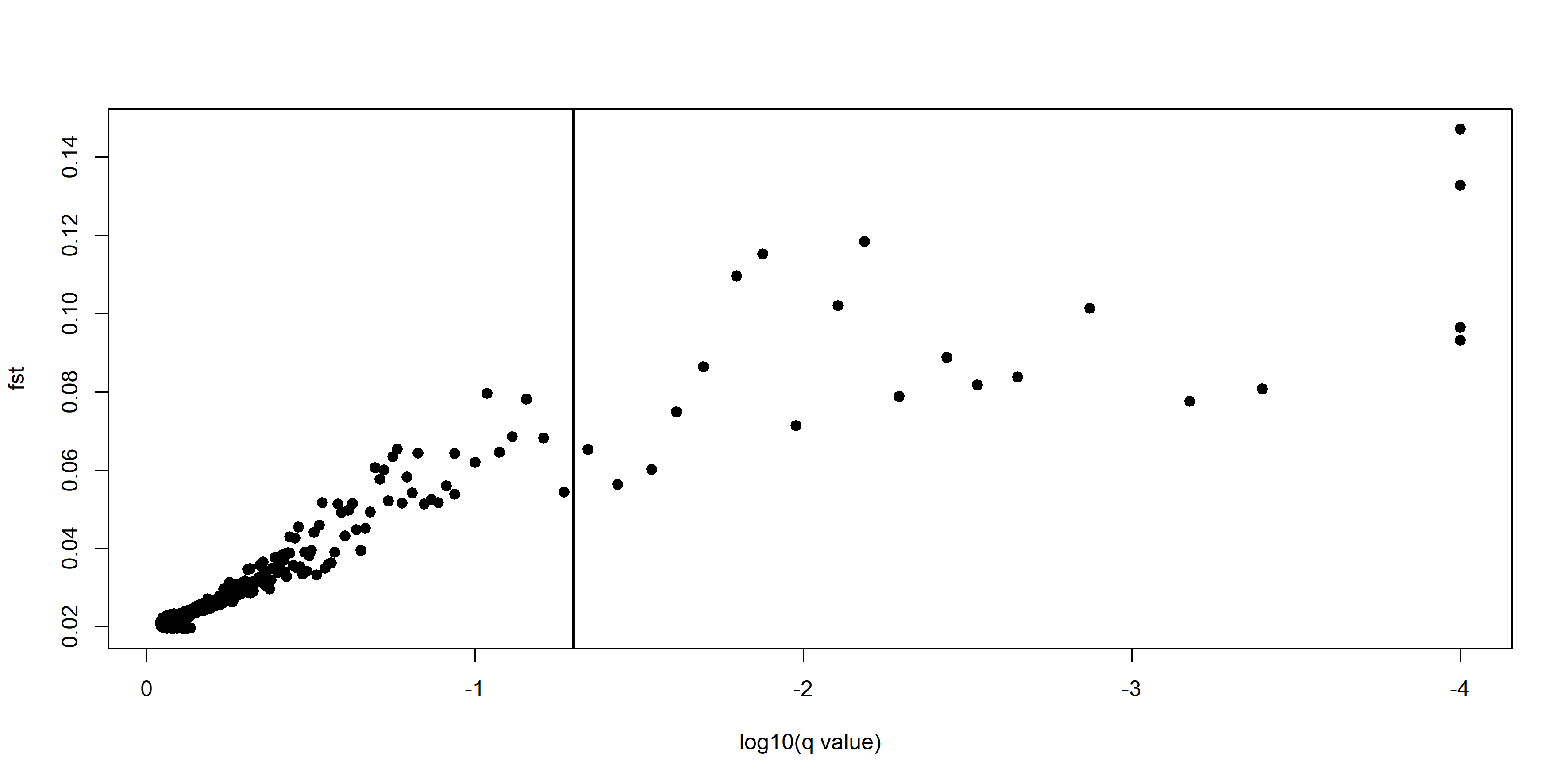


Supplementary figure 16: Detection of outlier loci in BayeScan, using a dataset of 493 Dipturus batis from 6 sampling locations, genotyped at 6,350 loci. Twenty-one loci under putative positive selection are shown to the right of the FDR threshold of 0.05 (vertical line).
